# SpdC, a novel virulence factor, controls histidine kinase activity in *Staphylococcus aureus*

**DOI:** 10.1371/journal.ppat.1006917

**Published:** 2018-03-15

**Authors:** Olivier Poupel, Caroline Proux, Bernd Jagla, Tarek Msadek, Sarah Dubrac

**Affiliations:** 1 Department of Microbiology, Biology of Gram-Positive Pathogens, Institut Pasteur, Paris, France; 2 ERL3526, CNRS, Paris, France; 3 Transcriptome and EpiGenome, BioMics, Center for Innovation and Technological Research, Institut Pasteur, Paris, France; 4 Center for Human Immunology, Center for Translational Science, Institut Pasteur, Paris, France; 5 Bioinformatics & Biostatistics HUB, Center of Bioinformatics, Biostatistics and Integrative Biology, Institut Pasteur, Paris, France; The University of Alabama at Birmingham, UNITED STATES

## Abstract

The success of *Staphylococcus aureus*, as both a human and animal pathogen, stems from its ability to rapidly adapt to a wide spectrum of environmental conditions. Two-component systems (TCSs) play a crucial role in this process. Here, we describe a novel staphylococcal virulence factor, SpdC, an Abi-domain protein, involved in signal sensing and/or transduction. We have uncovered a functional link between the WalKR essential TCS and the SpdC Abi membrane protein. Expression of *spdC* is positively regulated by the WalKR system and, in turn, SpdC negatively controls WalKR regulon genes, effectively constituting a negative feedback loop. The WalKR system is mainly involved in controlling cell wall metabolism through regulation of autolysin production. We have shown that SpdC inhibits the WalKR-dependent synthesis of four peptidoglycan hydrolases, SceD, SsaA, LytM and AtlA, as well as impacting *S*. *aureus* resistance towards lysostaphin and cell wall antibiotics such as oxacillin and tunicamycin. We have also shown that SpdC is required for *S*. *aureus* biofilm formation and virulence in a murine septicemia model. Using protein-protein interactions in *E*. *coli* as well as subcellular localization in *S*. *aureus*, we showed that SpdC and the WalK kinase are both localized at the division septum and that the two proteins interact. In addition to WalK, our results indicate that SpdC also interacts with nine other *S*. *aureus* histidine kinases, suggesting that this membrane protein may act as a global regulator of TCS activity. Indeed, using RNA-Seq analysis, we showed that SpdC controls the expression of approximately one hundred genes in *S*. *aureus*, many of which belong to TCS regulons.

## Author summary

*Staphylococcus aureus* is a major human pathogen, and has become a significant worldwide health concern due to the rapid emergence of antibiotic resistant strains. Like most bacteria, *S*. *aureus* adapts to its environment by adjusting its genetic expression through sensing and regulatory systems. We show here that the SpdC membrane protein is a novel virulence factor of *S*. *aureus*, controlling biofilm formation and pathogenesis. We show that SpdC interacts with the WalK histidine kinase to inhibit activity of the WalKR two-component system. SpdC also interacts with nine other histidine kinases of *S*. *aureus*, suggesting it acts as a pleiotropic global regulator.

## Introduction

Two-component systems (TCSs) are composed of a histidine kinase, usually membrane-bound and acting as an environmental sensor, which phosphorylates a coupled response regulator, often controlling gene transcription. Although these systems have been extensively studied and play an essential role in bacterial adaptation to the environment, the signal(s) to which they respond and additional factors positively or negatively controlling their activities remain mostly unknown. *Staphylococcus aureus*, a major human pathogen, causes diseases ranging from superficial cutaneous abscesses to life-threatening infections affecting all major organs [1]. *S*. *aureus* is also a commensal bacterium, colonizing approximately half the human population asymptomatically, essentially within the anterior nares [2]. In addition to its considerable arsenal of virulence factors, *S*. *aureus* must rapidly adapt to environmental conditions encountered during host colonization. Among the 16 TCSs encoded by the *S*. *aureus* genome [3], the SaeSR, AgrCA, and WalKR systems are particularly important for controlling virulence and innate immune evasion factors [4–8].

The WalKR system is the only *S*. *aureus* TCS shown to be essential for cell viability, suggesting that its activity may respond not only to environmental conditions but could also be controlled by intrinsic bacterial factors [9–11]. Indeed, it is becoming increasingly apparent that so-called two-component systems frequently involve additional proteins regulating the phosphorylation levels of the response regulator [12, 13]. These include accessory phosphatases such as CheZ, Spo0E or RapA, respectively dephosphorylating the *E*. *coli* CheY [14], *B*. *subtilis* Spo0A [15] and Spo0F [16] response regulators, or antikinases such as KipI and Sda that inhibit *B*. *subtilis* KinA [17, 18] and FixT which inhibits the *Sinorhizobium meliloti* FixJ kinase [19]. A sub-class of histidine kinases, known as intra-membrane sensing kinases [20], require the permease component of an associated ABC transporter for signal sensing, such as the *S*. *aureus* BraS [21] and GraS [22] kinases. Many TCS histidine kinases act as so-called bifunctional sensors, acting on their cognate response regulators both as kinases and phosphoprotein phosphatases [23]. Accordingly, several accessory proteins act by binding to the histidine kinase to inhibit its kinase activity or stimulate its phosphatase activity towards the response regulator. These include the PII protein acting on the NtrB kinase to control nitrogen assimilation by dephosphorylating NtrC [24], the *Streptococcus agalactiae* Abx1 Abi-domain membrane protein which interacts with the CovS histidine kinase to inhibit activity of the CovR response regulator [25], and the SaePQ protein complex, which stimulates the phosphatase activity of the SaeS histidine kinase in *S*. *aureus* [26].

In *Bacillus subtilis*, the WalK histidine kinase is thought to coordinate cell wall plasticity with cell division, with two membrane-bound accessory proteins, WalH and WalI, inhibiting WalK kinase activity [11, 27–29]. However, the WalH and WalI proteins of *S*. *aureus* share no significant sequence similarities with those of *B*. *subtilis* and their role is not as clear-cut. Indeed, although they are also membrane proteins and interact with the WalK kinase, WalH and WalI do not seem to play a major role in negatively controlling WalKR activity, suggesting that their functions have evolved [30].

In an effort to identify additional factors controlling the WalKR system, we showed that the *S*. *aureus* SpdC Abi-domain protein negatively affects WalK activity and expression of WalKR-regulated genes. We showed that SpdC, previously identified as playing a role in the display of surface proteins [31], forms a complex with the WalK histidine kinase and that the two membrane proteins preferentially localize at the division septum, suggesting that this interaction regulates WalK histidine kinase activity. The Δ*spdC* mutant displays a pleiotropic phenotype, including altered resistance towards compounds targeting the cell wall, as well as strongly diminished biofilm formation and virulence. Using RNA-Seq analysis, we showed that SpdC controls the expression of approximately one hundred genes in *S*. *aureus*. Indeed, SpdC activity appears to extend well beyond the WalKR system, since we have shown it also interacts with several other *S*. *aureus* histidine kinases suggesting it could be involved in controlling multiple regulatory/adaptive pathways.

## Results

### Expression of *spdC* is controlled by the WalKR system

**Fig 1:**
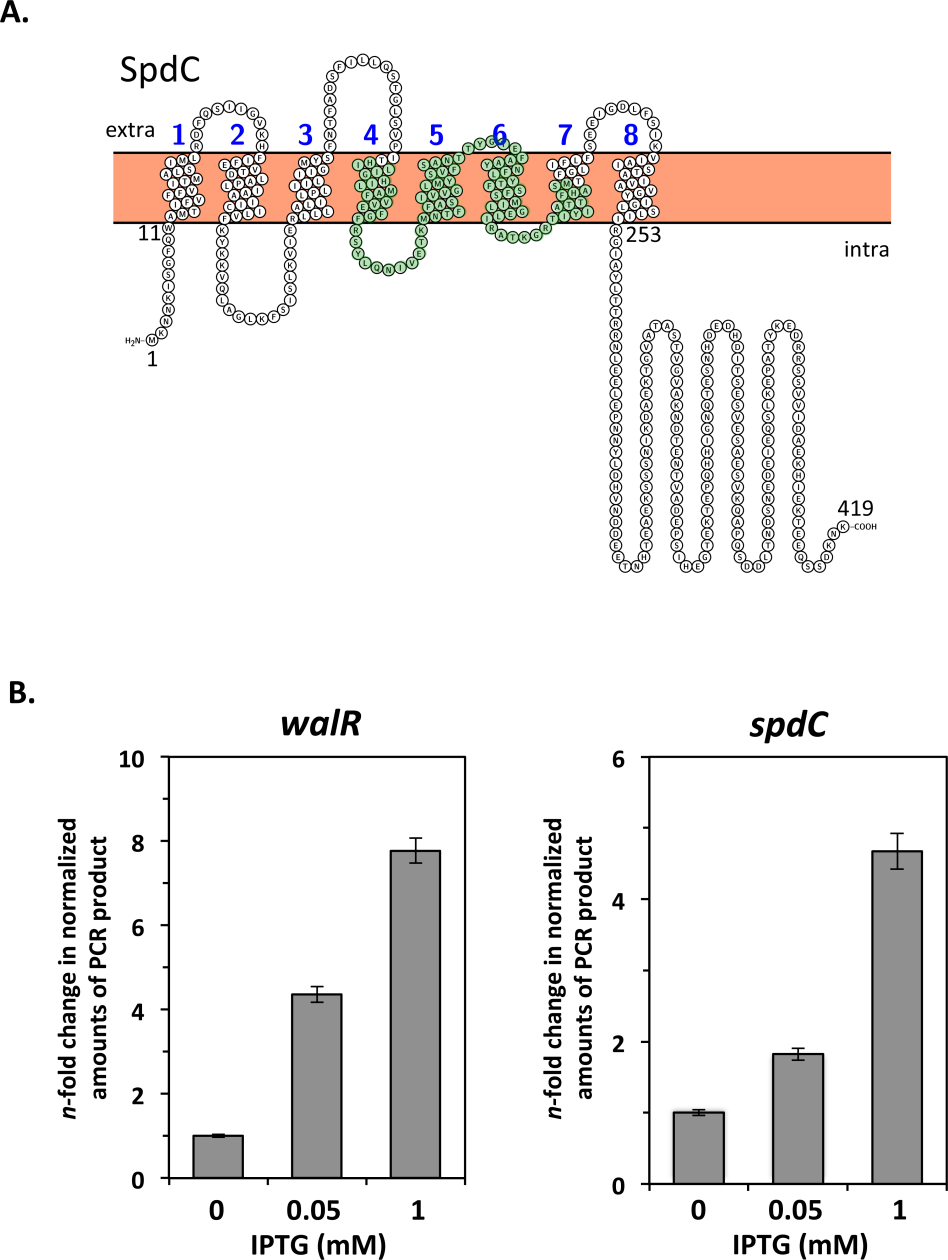
The WalKR two-component system controls expression of *spdC*, encoding a Abi-domain membrane protein. A. Predicted membrane topology of the SpdC protein generated using the Protter prediction tool (http://wlab.ethz.ch/protter/start/). The eight predicted transmembrane segments are numbered. Amino acids forming the putative Abi domain (based on Pfam database annotation) are shaded in green. B. Relative levels of *walR* and *spdC* transcripts were measured by qRT-PCR during growth of the HG001 Pspac*walRKHI* strain (ST1017). Bacteria were grown to exponential phase in TSB with or without increasing concentrations of IPTG to induce expression from the Pspac promoter. Expression levels were normalized using 16S rRNA as an internal standard and are indicated as *n*-fold change with respect to the control condition (absence of IPTG).

We previously performed an extensive transcriptome analysis in order to define the scope of the *S*. *aureus* WalKR regulon [6]. Our results showed that expression of the *spdC* gene (SAOUHSC_02611) was increased 3.5-fold in a *S*. *aureus* strain producing a constitutively active form of the WalR response regulator (D55E) [6]. SpdC, a membrane-anchored protein with 8 predicted transmembrane segments and an Abi domain (CAAX protease self-immunity), (Fig 1A), was previously reported as playing a role in the display of surface proteins such as protein A [31]. In order to confirm that *spdC* is a member of the WalKR regulon, we used quantitative real-time PCR (qRT-PCR) to measure its expression in a *S*. *aureus* strain where the *walRKHI* operon is placed under the control of the IPTG-inducible Pspac promoter [9]. Cells were grown overnight in TSB with 0.05 mM IPTG, and cultures were inoculated at OD_600nm_ = 0.05, with or without different IPTG concentrations (0.05 and 1 mM) to induce expression from the Pspac promoter. RNA samples were prepared from exponentially growing cells harvested at OD_600nm_ = 0.5, before cessation of growth of the culture lacking IPTG, and *walR* and *spdC* mRNA levels were measured by qRT-PCR. As shown in Fig 1B, *walR* transcription is increased approximately 4-fold when cells are grown with 0.05 mM IPTG and 8-fold at 1 mM IPTG. Under the same conditions, *spdC* expression followed that of *walR*, and was increased 2-fold and 5-fold with 0.05 or 1 mM IPTG, respectively (Fig 1B), confirming positive regulation by the WalKR system.

### SpdC is a pleiotropic regulator of gene expression

As shown above, the WalKR TCS controls *spdC* expression. In *Streptococcus agalactiae*, another bacterial Abi-domain protein, Abx1, has been shown to inhibit activity of the CovSR two-component system [25]. In order to determine whether SpdC has a regulatory role in *S*. *aureus*, we generated a Δ*spdC* mutant in strain HG001 and performed a comparative RNA-Seq analysis. The Δ*spdC* mutant did not display any gross morphological changes or growth defects. Indeed, although it had a slight lag during the first hour post inoculation, the growth rate and final OD_600nm_ were not significantly different from those of the parental strain (S1 Fig). Doubling times (http://www.doubling-time.com/compute.php) calculated during the exponential growth phase (S1 Fig, 90 min to 210 min) gave identical values of 32 min for both strains. The HG001 strain and Δ*spdC* mutant were grown in TSB until early exponential phase (OD_600nm_ = 1) and total RNA was extracted for RNA-Seq analysis (See Materials and Methods). We verified that *spdC* is well expressed under these conditions using a *lacZ* reporter fusion with the *spdC* promoter region (S2 Fig).

**Fig 2:**
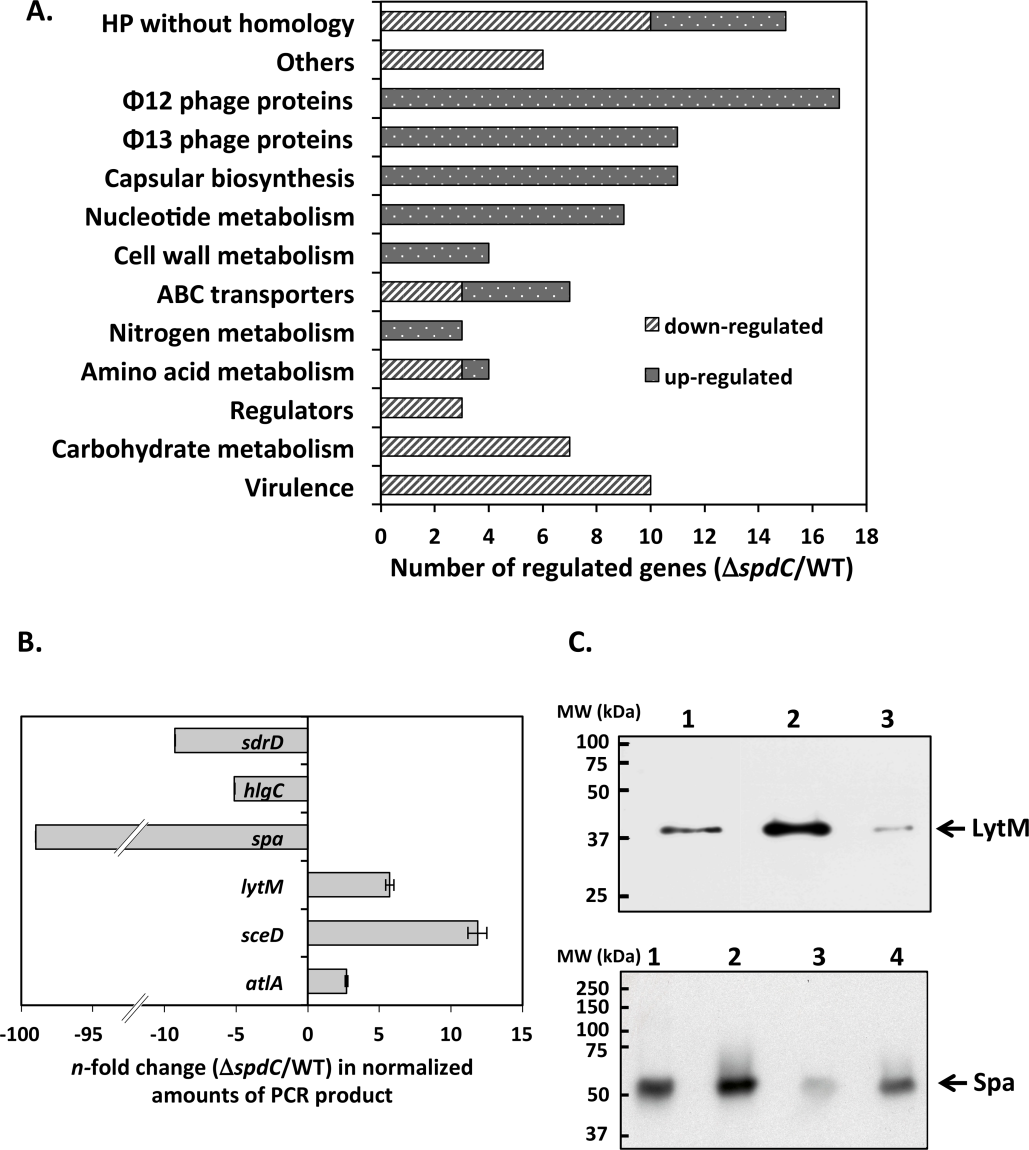
SpdC is involved in gene regulation. A. Ontological grouping of SpdC-regulated genes according to their annotated functions. Variations in gene expression were identified by RNA-Seq analysis of a Δ*spdC* strain compared to the HG001 parental strain grown in TSB until mid-exponential phase. B. qRT-PCR comparison of gene expression in a Δ*spdC* strain and the HG001 parental strain. Strains were grown in TSB until mid-exponential phase and RNA was extracted and treated as described in Materials and Methods. Expression levels were normalized using 16S rRNA as internal standard and presented as the *n-*fold change of the Δ*spdC* mutant strain compared to the HG001 parental strain. C. Western blot analysis of LytM (upper panel) and Spa (lower panel) production. Crude extracts (LytM) and cell wall extracts (Spa) were prepared from stationary phase cultures. Lanes: 1: Purified *Staphylococcus aureus* Protein A (50 ng) 2: HG001 strain; 3: Δ*spdC* mutant strain; 4: Δ*spdC*/pMK4Pprot-*spdC* complemented strain.

Three biological replicates were analyzed by RNA-Seq for each strain and the data presented as the mean fold-change. Using a value cut-off greater than 2 with a *P* value less than 0.05, we found that the expression of 42 genes was lowered in the Δ*spdC* mutant strain and that of 65 increased (Table 1). In order to perform a general analysis of the transcriptomic data we generated an ontological grouping of SpdC-regulated genes (Fig 2A). Among the genes positively controlled by SpdC, 10 are known virulence factors, suggesting that SpdC may influence *S*. *aureus* pathogenicity, and 11 are involved in capsular biosynthesis. The *S*. *aureus* capsule is known to impede phagocytosis and promote host colonization, however although the HG001 strain used in this study carries the serotype 5 capsule gene cluster, a missense mutation in the *cap5E* gene prevents capsular biosynthesis [32–34].

**Table 1:** Genes differentially expressed in the Δ*spdC* mutant compared to the parental HG001 strain.

**SpdC-activated genes**			
Id^(a)^	Gene name/function	Fold change^(b)^	Also regulated by^(c)^
Virulence			
00069*	*spa*	-59.69	VraSR, WalKR
02709*	*hlgC*	-5.53	**SaeSR, WalKR**
02710*	*hlgB*	-4.97	ArlSR, **GraSR, SaeSR, WalKR**
00545*	*sdrD*	-4.19	**ArlSR, GraSR, AirSR**
02169*	*chp*	-2.86	**SaeSR, GraSR, WalKR**
01939*	*splC*	-2.16	BraSR, **SaeSR, WalKR**
01941*	*splB*	-2.49	ArlSR, **SaeSR, WalKR**
02171*	*sak*	-2.03	**SaeSR**
01112*	formyl peptide receptor-like 1 inhibitory prot.	-2.23	**SaeSR, WalKR**
01110*	fibrinogen-binding prot.	-2.04	**SaeSR, WalKR**
Regulators			
00070*	*sarS*	-2.55	WalKR, **GraSR, BraSR**
00961	*comK*	-2.12	
01879	*rot*	-2.03	**ArlSR, GraSR,**
ABC transporters			
02820	ABC transporter ATP binding prot.	-5.94	VraSR,**GraSR, BraSR**
02821	ABC transporter permease	-5.27	**ArlSR, GraSR, BraSR**
01389	PstS phosphate ABC transporter	-3.39	
Amino acid metabolism			
01452	alanine dehydrogenase	-4.29	**ArlSR, SaeSR, GraSR, BraSR,**
01451*	threonine dehydratase	-5.32	**ArlSR, SaeSR, GraSR, BraSR, WalKR**
01450	putative AA permease	-4.42	
Carbohydrate metabolism			
00051	1-phosphatidylinositol phosphodiesterase	-3.27	
00187	formate acetyl transferase	-2.92	**SaeSR**
00188*	pyruvate formate-lyase 1 activating enzyme	-3.04	**SaeSR, WalKR**
00608	alcohol dehydrogenase	-2.75	**SaeSR**
00113	bifunctional acetaldehyde-CoA/alcohol dehydrogenase	-2.72	BraSR, **GraSR**
02830*	D-lactate dehydrogenase	-2.20	GraSR, WalKR
02922	L-lactate dehydrogenase	-2.11	**BraSR**
Other			
01448	putative transporter/multidrug resistance prot.	-3.31	**ArlSR, GraSR, SaeSR**
02108	FtnA ferritin	-2.21	**SaeSR, GraSR**
02862*	*clpC*	-2.15	WalKR
02127	staphopain thiol protease	-2.05	
00625	monovalent cation/H+ antiporter subunit A	-2.02	
02941	anaerobic ribonucleoside-triphosphate reductase activating prot. (*nrdG)*	-2.08	
HP without homology			
02781		-2.85	GraSR
00966		-2.82	
00203		-2.61	**BraSR**
01123		-2.52	
02782		-2.51	
00094*		-2.31	WalKR, **BraSR**
02294		-2.20	
02973		-2.17	
02842		-2.14	GraSR, WalKR
01798		-2.10	**BraSR**
**SpdC-repressed genes**			
Id^(a)^	Gene name/function	Fold change^(b)^	Also regulated by^(c)^
Cell wall metabolism			
02333*	*sceD*	10.80	**GraSR, WalKR**
02576*	*ssaA*	5.64	**GraSR, WalKR, SaeSR**
00248*	*lytM*	3.81	**WalKR**
00994*	*atlA*	2.21	**GraSR, WalKR**
ABC transporters			
02430	ABC transporter substrate-binding prot.	2.69	**GraSR**
02153	ABC-2 transporter family prot.	2.20	
02154	ABC transporter ATP-binding prot.	2.19	
02152	ABC transporter ATP-binding prot.	2.13	
Φ12 phage proteins			
01535	HP	4.00	
01573	HP	3.02	
01538	phage terminase large subunit	2.93	
01536	scaffolding protease	2.66	
01515	peptidoglycan hydrolase	2.66	
01516	holin prot.	2.63	
01530	HP	2.60	
01532	SLT orf 110-like prot.	2.50	
01570	PVL orf 37-like prot.	2.43	
01566	phi APSE P51-like prot.	2.43	
01531	SLT orf 123-like prot.	2.42	
01519	SLT orf 129-like prot.	2.37	
01520	SLT orf 488-like prot.	2.33	
01571	SLT orf 71-like prot.	2.28	
01533	HP	2.17	
01527	HP	2.13	
01529	major tail prot.	2.03	
Φ13 phage proteins			
02234	repressor-like prot.	4.83	
02233	phi PVL orf 32-like prot.	3.77	
02221	HP	3.19	
02220	phi ETA orf 18-like prot.	2.71	
02228	HP	2.61	
02211	phi PVL orf 50-like prot.	2.58	
02225	HP	2.26	
02219	phi ETA orf 20-like prot.	2.21	
02216	phage DnaC-like prot.	2.20	
02223	phi PVL orf 39-like prot.	2.06	
02222	HP	2.05	
Nucleotide metabolism			
01172*	PyrE orotate phosphoribosyltransferase	8.02	**SaeSR, WalKR**
01171*	orotidine 5'-phosphate decarboxylase	7.99	**SaeSR, WalKR**
01170*	CarB carbamoyl phosphate synthase large subunit	7.04	**SaeSR, WalKR, BraSR**
01166*	*pyrB*	3.53	**SaeSR, WalKR**
01017	*purH*	2.19	**GraSR, SaeSR**
02558	UreA urease subunit gamma	2.14	ArlSR, GraSR , SaeSR
01016	phosphoribosylglycinamide formyltransferase	2.12	**GraSR, SaeSR**
01018	phosphoribosylamine—glycine ligase	2.07	**GraSR, SaeSR**
01015	phosphoribosylaminoimidazole synthetase	2.00	**GraSR, SaeSR**
Nitrogen metabolism			
02685	nitrite reductase transcriptional regulator NirR	2.48	**BraSR**
02684	assimilatory nitrite reductase [NAD(P)H] large subunit	2.03	**VraSR**
02681	nitrate reductase subunit alpha	2.03	**BraSR**
Amino acid metabolism			
01367	anthranilate synthase component II	2.49	
Capsular biosynthesis			
00116	Cap5C	2.40	
00117	Cap5D	2.37	BraSR, **ArlSR**
00122	Cap5I	2.32	BraSR, **GraSR**
00114	Cap5A	2.28	**ArlSR**
00115	Cap5B	2.28	
00128	Cap5O	2.19	
00120	Cap5G	2.17	BraSR
00121	Cap5H	2.13	BraSR, **GraSR**
00123	Cap5J	2.12	**GraSR**
00125	Cap5L	2.09	BraSR
00126	Cap5M	2.03	
HP without homology			
01173		3.70	**SaeRS**
01583		2.75	BraSR, **SaeSR**
01084		2.71	
01584		2.54	
02863		2.06	

*: Genes also regulated by the WalKR system

(a): Id indicates the *S*. *aureus* NCTC 8325 genome sequence SAOUHSC _ annotation.

(b): Fold change was determined as the ratio of the signal values between the Δ*sdpC* strain and the parental strain. Negative values indicate the gene is less expressed in the Δ*spdC* mutant strain and positive values that it is more highly expressed in the mutant strain. Only fold changes >2 with a *P* value <0.05 for the three biological replicates were considered.

(c): SpdC-regulated genes identified by RNA-Seq data analysis were compared with publicly available transcriptome analyses of *S*. *aureus* TCS mutants (http://www.satmdorg/) and TCSs with which SpdC was shown to interact are listed. Names in bold indicate positive regulation by the TCS in question as opposed to negative regulation.

Expression of several *S*. *aureus* prophage genes was also increased in the Δ*spdC* mutant strain: 11 for phage Φ13 and 17 for Φ12, (Table 1).

### SpdC negatively controls WalKR regulon genes

As shown above, *spdC* expression is controlled by the WalKR system. The RNA-Seq data analysis of SpdC-regulated genes reveals that 25 of these belong to the WalKR regulon (indicated by an asterisk in Table 1). In particular, the expression of 4 WalKR-dependent cell wall hydrolase genes (*sceD*, *ssaA*, *lytM* and *atlA*) is increased in the Δ*spdC* mutant, suggesting that SpdC negatively controls WalKR activity (Table 1). It is interesting to note that among the genes positively controlled by SpdC, many are also WalKR-activated genes (Table 1). All of these are classified as virulence genes, however they are not preceded by the WalR consensus binding site, and we have previously shown that several of these are not directly regulated by the WalKR system but through the SaeRS two-component system instead [6].

We used qRT-PCR to verify SpdC-dependent regulation for 3 positively (*spa*, *hlgC*, *sdrD*) and 3 negatively (*atlA*, *sceD*, *lytM*) controlled genes, in the Δ*spdC* strain compared to the HG001 parental strain, grown under the same conditions as for the RNA-Seq analysis (Fig 2B). We observed a perfect correlation with the RNA-Seq data: expression of the *spa* gene encoding protein A was very strongly lowered in the Δ*spdC* mutant strain (about one hundred-fold), while *sdrD* and *hlgC* expression levels were 5- to 10-fold less. The *atlA*, *sceD* and *lytM* cell wall hydrolase genes were more highly expressed in the absence of SpdC (2-, 12- and 5-fold, respectively) in agreement with the RNA-Seq analysis (Fig 2B).

In order to confirm SpdC-dependent regulation at the protein level, we chose two genes that were positively or negatively controlled by SpdC, *spa* and *lytM*, respectively, and performed Western blot analyses. Whole cell extracts were prepared from cultures of strains HG001, the Δ*spdC* mutant and the complemented mutant strain (Δ*spdC*/pMK4Pprot-*spdC*) and subjected to SDS-PAGE and immunoblotting. As shown in the top panel of Fig 2C, LytM levels are higher in the Δ*spdC* strain compared to the parental strain (lane 2), and in the complemented strain the LytM level is reduced to a level lower than in the parental strain (lane 3), likely reflecting the higher production of SpdC in the complemented strain. Indeed, under these conditions, *spdC* mRNA levels were increased more than 100-fold as measured by qRT-PCR as compared to the parental HG001 strain.

For studying levels of protein A, known to be covalently anchored to the cell wall (LPxTG sortase motif), identical quantities of cell wall fractions of the HG001 parental strain, Δ*spdC* deletion mutant, and complemented strain (Δ*spdC*/pMK4Pprot-*spdC*) were subjected to SDS/PAGE and compared by Western blot. As expected, protein A levels were significantly lower in the Δ*spdC* mutant than in the parental and complemented strains (Fig 2C, lower panel), in agreement with the RNA-Seq and qRT-PCR results.

These data indicate that SpdC is involved in controlling gene expression through potential interactions with regulatory systems, and the WalKR two-component system in particular.

### SpdC is localized at the division septum and interacts with the WalK histidine kinase

**Fig 3:**
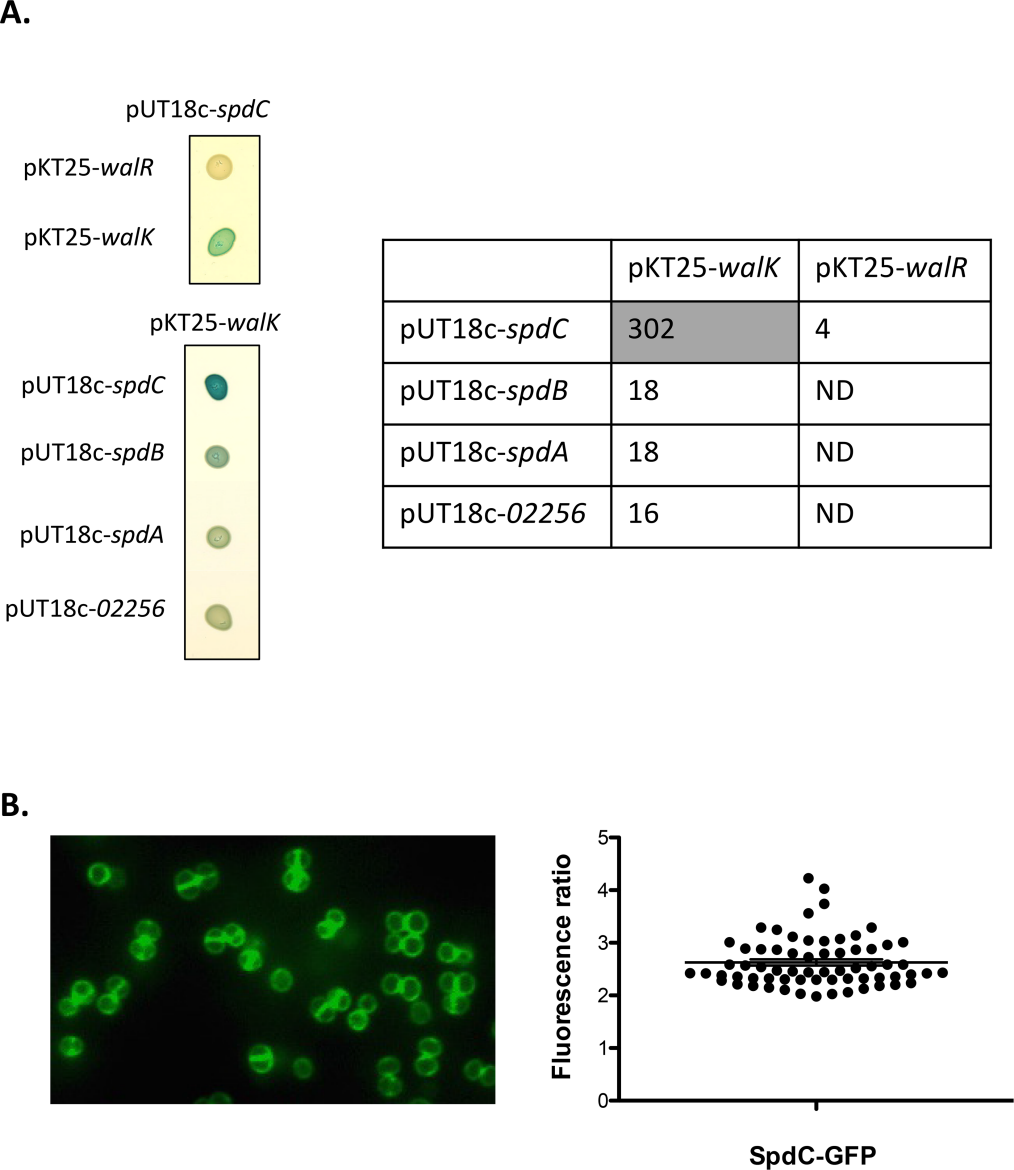
The SpdC membrane protein forms a complex with the WalK histidine kinase at the division septum. A: The SpdC protein was tested for pairwise interactions with the WalK histidine kinase and the WalR response regulator using the BACTH complementation assay by translationally fusing the full length corresponding coding sequences with those of the T25 or T18 adenylate cyclase domains (upper left panel). Specificity of the SpdC-WalK complex was confirmed by testing interactions with three other *S*. *aureus* Abi-domain proteins: SpdB, SpdA and SAOUHSC_02256 (lower left panel). Cultures of *E*. *coli* DHT1 strains co-transformed with the indicated plasmid combinations were spotted on LB agar plates with X-Gal as an indicator of β-galactosidase activity (see Materials and Methods). Quantitative β-galactosidase activity assays for each strain are indicated in the right panel (expressed in Miller Units). The shaded cell indicates significant β-galactosidase activity resulting from positive protein-protein interactions. ND: Not determined. B: *S*. *aureus* HG001 cells producing a fluorescent SpdC-GFP protein fusion (HG001/pOLSA-*spdC*) were grown in TSB with 0.25 μM CdCl_2_ and observed in exponential phase by fluorescence microscopy. Fluorescence ratios (septum/lateral membrane) were quantified for two biological replicates (33 cells from each) using ImageJ software and plotted using GraphPad Prism. The horizontal line indicates the mean fluorescence ratio and the error bars represent the SEM.

As shown above, we have uncovered a regulatory link indicating that SpdC negatively controls activity of the WalKR two-component system, strongly suggesting that the proteins interact. In order to test possible interactions between SpdC and the WalKR proteins, we used the bacterial adenylate cyclase two-hybrid system (BACTH) [35]. We fused the full-length membrane-bound WalK histidine kinase or the WalR cytoplasmic response regulator to the C-terminal domain of the T25 subunit of the *Bordetella pertussis* adenylate cyclase and full-length SpdC to the C-terminal domain of the T18 subunit, using plasmids pKT25 and pUT18c respectively. To probe putative interactions, *E*. *coli* strain DHT1 was co-transformed with combinations of the pKT25 and pUT18c derivatives carrying the translational fusions. Upon protein-protein interactions, the close proximity between the T18 and T25 subunits restores adenylate cyclase activity, leading to cAMP synthesis and activation of the lactose operon. Interactions were tested both by spotting the resulting strains on LB plates containing X-Gal and by measuring β-galactosidase activity. To determine pair-wise interactions, we chose a cut-off value of 100 Miller Units as indicating a positive interaction between the protein fusions. As shown in Fig 3A, strong β-galactosidase activity was only observed for the plasmid combination co-producing the membrane anchored proteins SpdC and WalK while no interactions between SpdC and the cytoplasmic regulator WalR could be detected. SpdC is annotated as an Abi domain protein (CAAX protease self-immunity) in genome databases. The *S*. *aureus* HG001 genome encodes 4 Abi domain proteins, three of which, SpdA (SAOUHSC_01900), SpdB (SAOUHSC_02587), and SpdC, have been reported as being involved in surface protein display, whereas the fourth (SAOUHSC_02256) has no known function [31]. In order to test whether WalK also interacts with the other three Abi domain proteins, we constructed translational fusions for the remaining Abi proteins with the T18 domain of adenylate cyclase. As shown in Fig 3A, the combinations of SpdA, SpdB or SAOUHSC_02256 with WalK did not generate significant levels of β-galactosidase activity, demonstrating that SpdC is the only *S*. *aureus* Abi domain protein specifically interacting with WalK.

We have previously shown that the *S*. *aureus* WalK histidine kinase is mainly localized at the division septum [30]. Since SpdC and WalK interact, we studied the subcellular localization of SpdC in *S*. *aureus* by constructing a translational fusion with the GFP fluorescent protein using the pOLSA vector (See Materials and Methods), under the control of the cadmium-inducible P_*cad*_ promoter. The resulting plasmid, pOLSA-*spdC* was then introduced into the HG001 strain. Expression of the gene fusion was induced by addition of CdCl_2_ (0.25 μM), cells were harvested during exponential growth (OD_600nm_ = 1.5) and observed by fluorescence microscopy. As shown in Fig 3B, SpdC is preferentially localized at the division septum, with a mean septum/membrane fluorescence ratio of around 2.6. Taken together, our results indicate that SpdC and WalK interact and are localized at the division septum.

### SpdC is involved in *S*. *aureus* resistance against compounds targeting the cell envelope

As shown above, the expression of several cell wall hydrolase genes is significantly increased in the Δ*spdC* mutant strain, suggesting that sensitivity to compounds targeting the cell wall might also be affected. We followed bacterial lysis during incubation in the presence of a non-anionic detergent, Triton X-100, thought to trigger cell lysis by favoring endogenous autolysin activity [36]. However, Triton X-100 induced lysis for the HG001 and Δ*spdC* mutant strains was not significantly different (S3 Fig).

**Fig 4:**
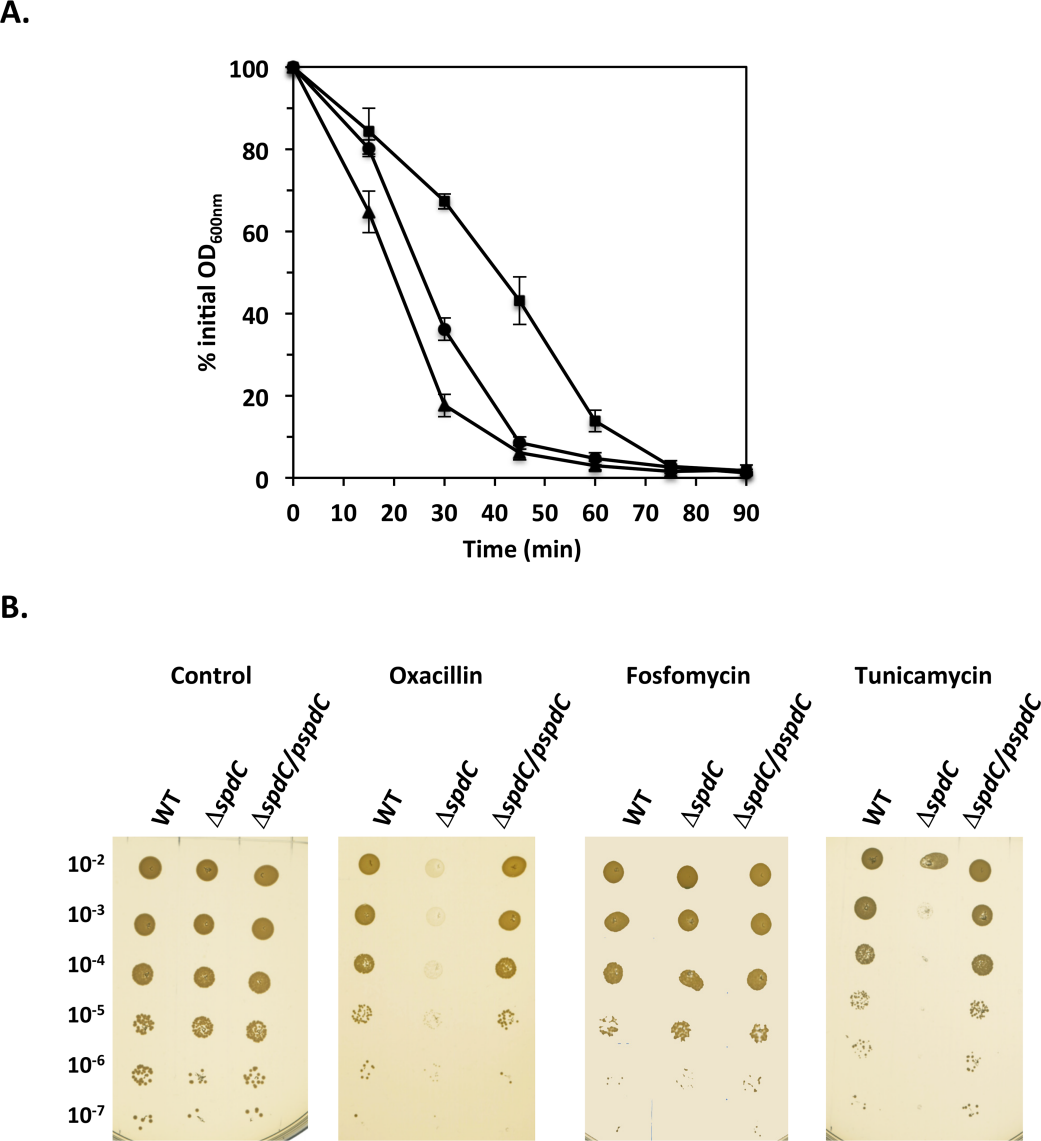
SpdC impacts cell wall homeostasis. A. The Δ*spdC* mutant displays increased resistance to lysostaphin-induced lysis. Cells were grown in TSB until mid-exponential phase, harvested and incubated in PBS with lysostaphin (200 ng/ml) with aeration at 37°C. Bacterial lysis was measured by monitoring OD_600nm_ over time. Results are shown as the mean and standard deviation of three independent assays. HG001 parental strain (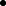); Δ*spdC* mutant (■); Δ*spdC*/pMK4Pprot-*spdC* complemented strain (▲). B. The absence of SpdC leads to sensitivity to oxacillin and tunicamycin. Dilution series of the HG001, Δ*spdC* and Δ*spdC*/ pMK4Pprot-*spdC* strains on TSA plates with or without antibiotics. Oxacillin: 0.1 μg/ml; fosfomycin: 4 μg/ml; tunicamycin: 1 μg/ml.

We also tested sensitivity to lysostaphin, a glycyl-glycine endopeptidase that hydrolyzes the peptidoglycan pentaglycine interpeptide crossbridge, leading to cell lysis [37]. The HG001, Δ*spdC* mutant and complemented strains were grown in TSB until OD_600nm_ ≈ 1 and cells were then harvested and resuspended in PBS in the presence of lysostaphin. As shown in Fig 4A, the Δ*spdC* mutant was less sensitive to lysostaphin-induced lysis than the parental HG001 and complemented Δ*spdC* strains, suggesting that the absence of SpdC leads to cell wall modifications.

We then tested sensitivity to antibiotics targeting the cell wall. As shown in Fig 4B the Δ*spdC* mutant displayed increased sensitivity to the β-lactam antibiotic oxacillin, whereas the parental and complemented strains were able to grow at the concentration tested (0.1 μg/ml). No difference in sensitivity between the strains was seen using fosfomycin, an antibiotic inhibiting MurA, which catalyzes the very first step of peptidoglycan biosynthesis (Fig 4B). These results suggested that the Δ*spdC* mutant strain may either be affected in the later steps of peptidoglycan biosynthesis or may exhibit a cell wall structure modification leading to a difference in accessibility of antibiotics acting extracellularly. Wall teichoic acids (WTAs) are anionic sugar rich cell surface polymers that can alter accessibility to the cell wall. We therefore tested resistance to tunicamycin, an antibiotic targeting biosynthesis of WTAs. As shown in Fig 4B the Δ*spdC* mutant strain was highly sensitive to tunicamycin, in contrast to the parental and complemented strains.

Taken together, these results suggest that the *S*. *aureus* cell envelope structure is altered in the absence of SpdC.

### SpdC is required for biofilm formation

**Fig 5:**
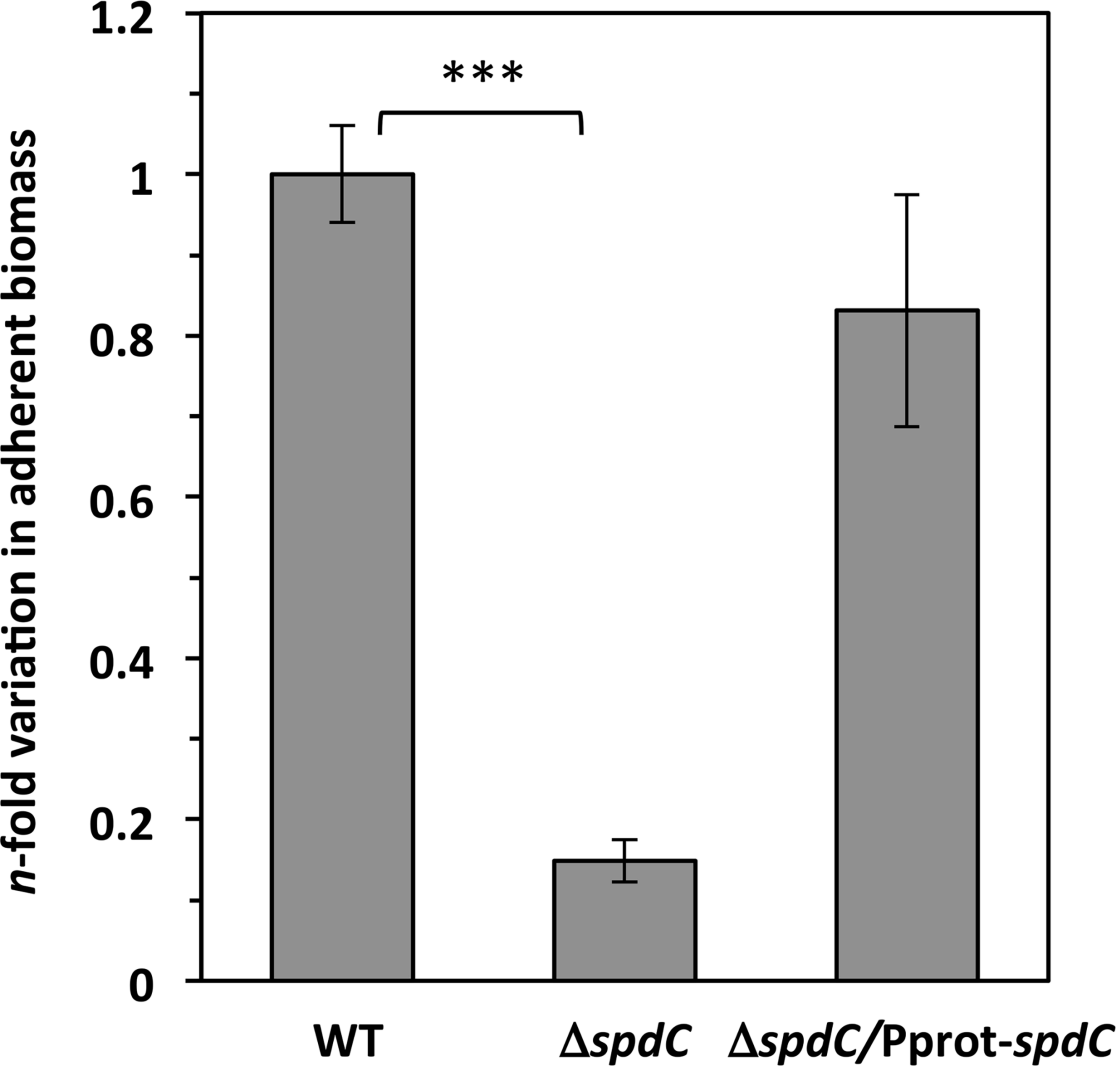
SpdC is required for biofilm formation. Biofilm assays were performed by growing cells in static cultures in PVC microtiter plates (TSB plus 0.75% glucose and 3.5% NaCl). Plates were incubated at 37°C for 24h and adherent biomass was quantified as described in Materials and Methods. Data were normalized to the OD_600nm_ of each culture and indicated as the *n-*fold change of the Δ*spdC* mutant strain compared to the HG001 parental strain. ***, *P*<0.0005 (Student’s *t*-test).

Since our results indicate that the Δ*spdC* mutation may modify the *S*. *aureus* cell surface, we tested whether the absence of SpdC may have an effect on biofilm formation. Strains were grown statically in TSB, supplemented with glucose and NaCl, for 24 h at 37°C in PVC microplates. As shown in Fig 5, biofilm formation was strongly decreased in the absence of SpdC (approximately 7-fold). Complementation of the Δ*spdC* mutant with the pMK4Pprot-*spdC* plasmid restored biofilm formation to levels comparable to those of the parental HG001 strain (Fig 5). These results are consistent with a modification of the *S*. *aureus* cell surface in the absence of SpdC, which could influence resistance against antimicrobial compounds targeting cell surface structures as well as the capacity to form biofilms. In order to determine which biofilm component is affected, biofilm detachment experiments were carried out (S4 Fig) by treatment with proteinase K, DNaseI and sodium metaperiodate (a carbohydrate-modifying agent). Under our conditions, biofilm production was lowered three-fold after treatment with DNaseI, and more than 10-fold when treated with proteinase K, but not significantly modified after treatment with sodium metaperiodate. Thus, biofilms formed under our conditions by the HG001 parental strain are essentially protein-based, and, to a lesser extent, due to extracellular DNA. We observed reduced biofilm formation for the Δ*spdC* mutant even after DNAseI treatment, but not after proteinase K treatment (S4 Fig), suggesting that SpdC affects the production of proteins important for biofilm formation.

### SpdC is a novel virulence factor

**Fig 6:**
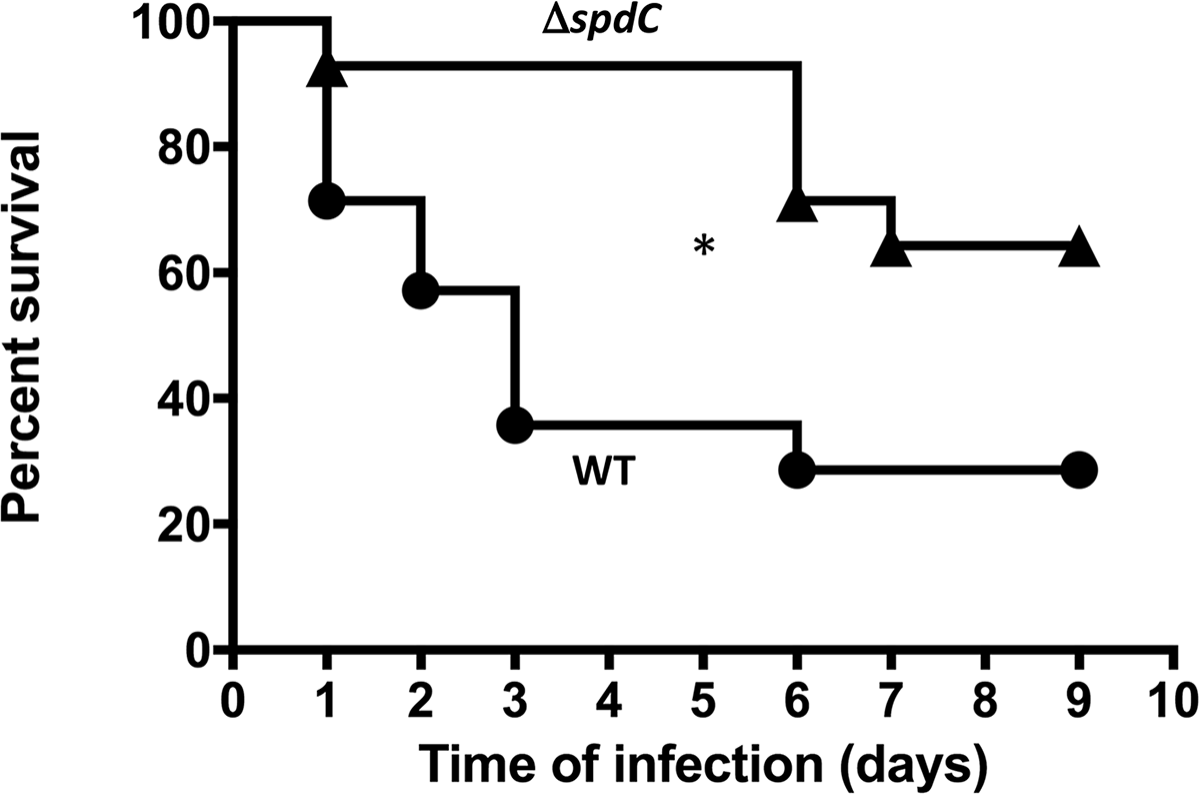
SpdC is a novel *S*. *aureus* virulence factor. Kaplan-Meier survival curves of *RjOrl*:*SWISS* mice infected with either the HG001 parental strain (circles) or the Δ*spdC* strain (triangles) by i.v. route (5.10^7^ cfu/injection). A total of 14 mice were used in each group in two independent experiments. *, *P*<0.05 (Wilcoxon test).

Cell surface modifications are known to impact virulence. Likewise, the capacity to form robust biofilms favors bacterial colonization of the host. Additionally, our RNA-Seq analysis revealed that the expression of at least 10 genes directly involved in bacterial virulence upon infection was lowered in the Δ*spdC* mutant, strongly suggesting that SpdC plays a role in virulence. We used a murine sepsis model to compare virulence of the HG001 and Δ*spdC* strains. SWISS mice were infected intravenously with 5.10^7^ cfu and mortality was monitored over 9 days post infection. As shown in Fig 6 virulence of the Δ*spdC* mutant was strongly diminished. Indeed, following infection with the HG001 parental strain, significant mortality occurred in the first 3 days post-infection (greater than 60%), whereas only a single mouse out of 14 died within five days after infection with the Δ*spdC* mutant. After the sixth day, a moderate mortality was observed for the group infected with the Δ*spdC* mutant, with only 36% mortality at the end of the assay (compared to 72% mortality for mice infected with the parental HG001 strain). This significant difference indicates that SpdC is a novel virulence factor in *S*. *aureus*.

### SpdC interacts with several histidine kinases through their transmembrane domains

**Fig 7:**
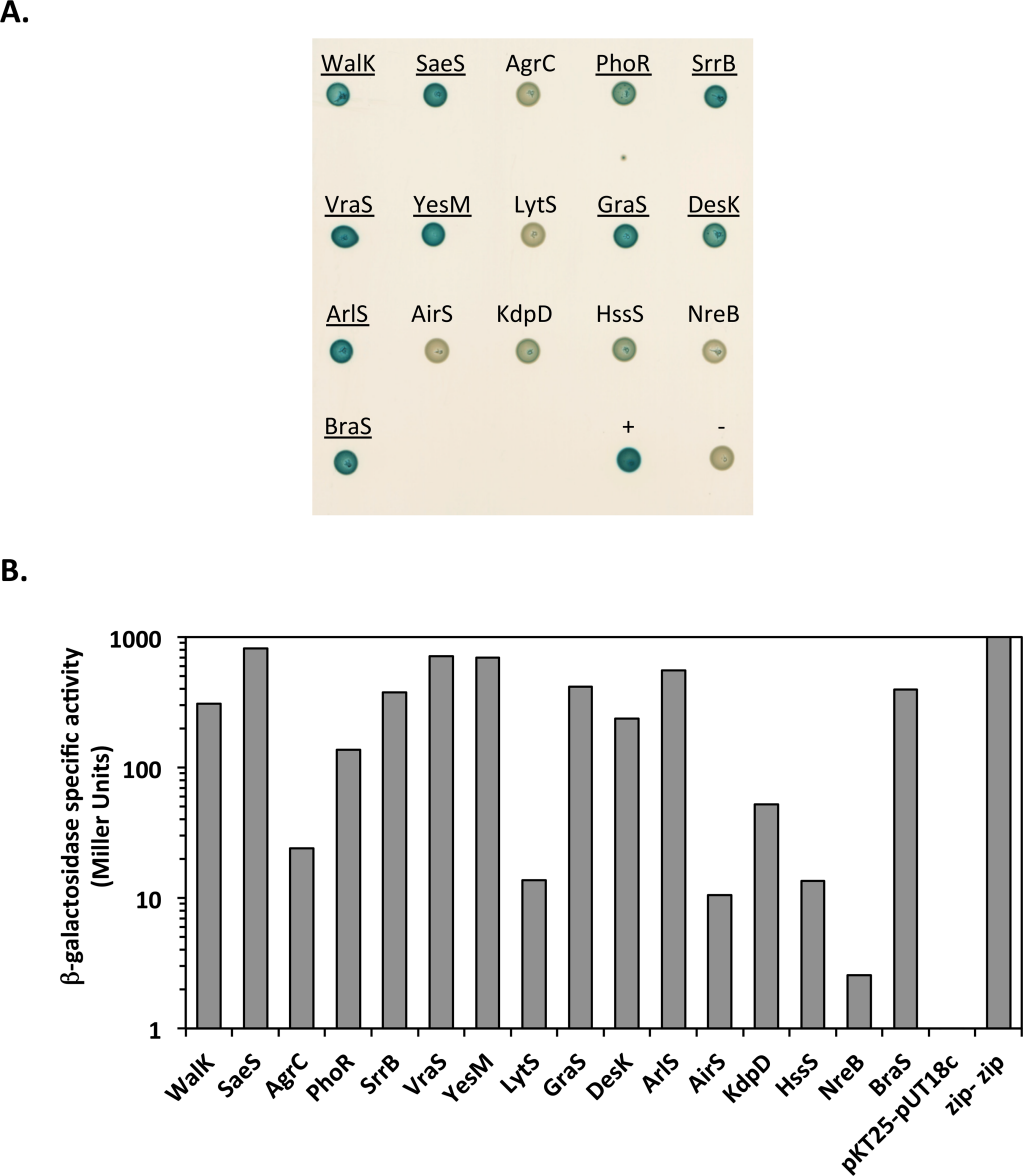
Protein interaction network between SpdC and *S*. *aureus* histidine kinases. The BACTH assay was used to test protein-protein interactions between SpdC and each of the *S*. *aureus* TCS histidine kinases. A. DHT1 *E*. *coli* strains co-transformed with each combination of plasmids (pUT18c-*spdC* and pKT25 derivatives carrying the genes for the designated kinases) were spotted on LB agar plates with X-gal as an indicator of β-galactosidase activity. Histidine kinases showing a positive result for interaction with SpdC are underlined. B. β-galactosidase activity of DHT1 strains co-transformed with the pUT18c-*spdC* and pKT25 derivatives carrying each of the histidine kinase encoding genes. β-galactosidase activity (Miller Units) of the strain carrying the empty vectors (pKT25-pUT18c) was arbitrarily fixed at 0 and that of the positive control (pKT25zip-pUT18czip) at 1000.

As shown above, SpdC localizes at the division septum and interacts with the WalK histidine kinase, negatively controlling WalKR activity. However, many of the SpdC-regulated genes identified by RNA-Seq do not belong to the WalKR regulon, but are known to be controlled by other two-component systems (Table 1) suggesting SpdC may interact with other TCS regulatory pathways. The *S*. *aureus* HG001 genome encodes 16 two-component systems [33] and we constructed translational fusions for each of the histidine kinase genes with the carboxy-terminal region of the adenylate cyclase T25 domain. Each of the resulting plasmids was co-transformed in combination with the pUT18c-*spdC* plasmid into *E*. *coli* strain DHT1. As shown in Fig 7, in addition to WalK, we detected interactions between SpdC and nine additional histidine kinases: YesM, GraS, SaeS, DesK, ArlS, SrrB, PhoR, VraS, and BraS. These interactions appear to be specific, since no interactions were detected between SpdC and the remaining six histidine kinases, as shown in Fig 7 (LytS, AirS, AgrC, KdpD, HssS, and NreB).

Among the genes positively regulated by SpdC (Table 1), the expression of at least fifteen is also activated by the SaeSR TCS, including the *spl* operon, *sak*, the *hlgBC* operon and *chp* [7, 38–40]. This suggests that SpdC activates the SaeSR TCS, in contrast to its role in negatively controlling activity of the WalKR system.

Histidine kinases have different combinations of signaling domains such as HAMP or PAS domains [41] in addition to the conserved H, N, G1, F and G2 boxes of the phosphoacceptor/dimerization (HisKA) and catalytic (HATPase_C) domains [42–44]. We have shown that SpdC interacts with 10 of the 16 *S*. *aureus* histidine kinases, which do not share any strong amino acid sequence similarities other than the conserved histidine kinase HisKA and HATPase_C domains. Since SpdC interacts with some, but not all of the *S*. *aureus* histidine kinases, this protein-protein contact must involve some other domain.

**Fig 8:**
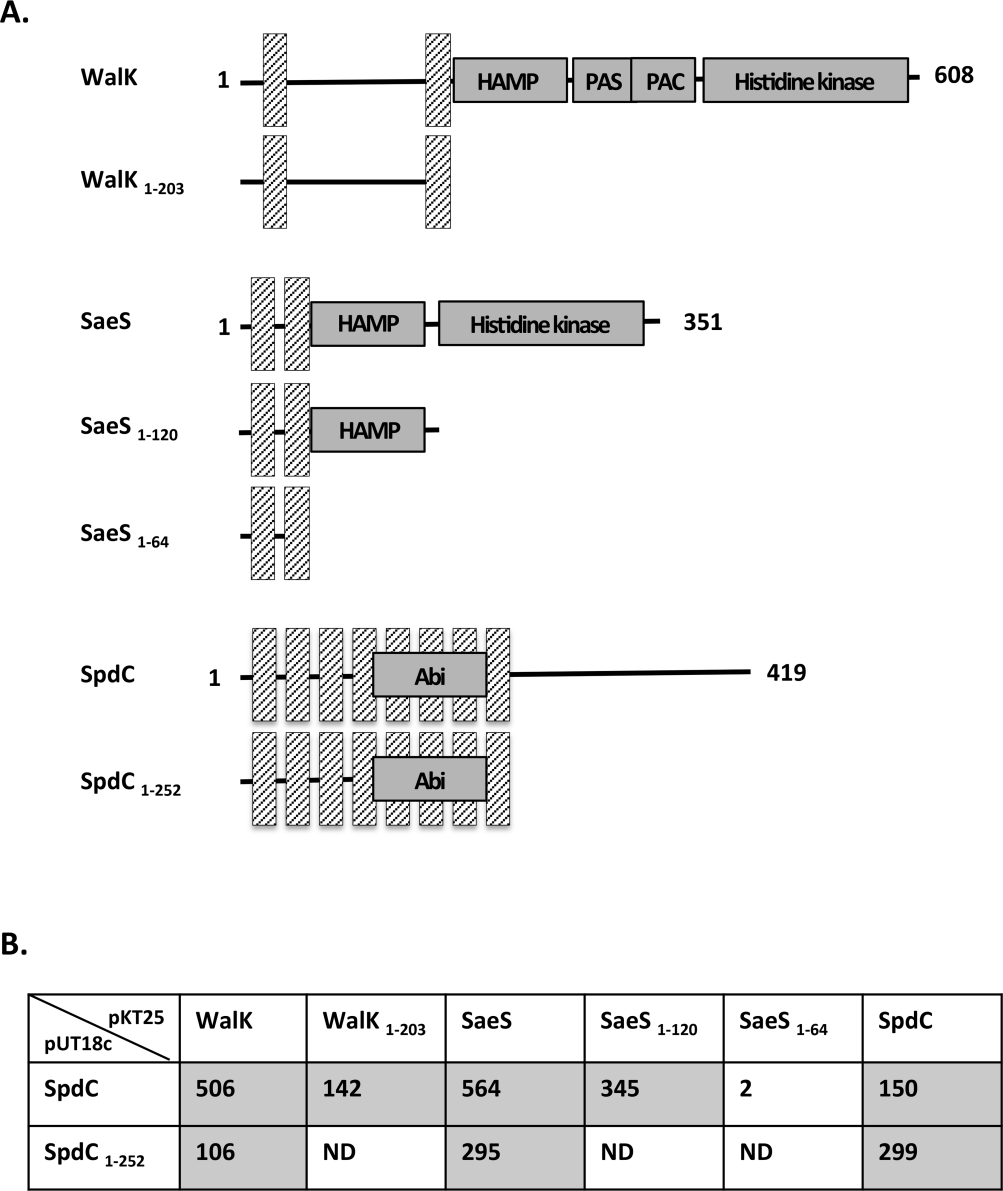
Domains involved in interaction between SpdC and the WalK and SaeS histidine kinases. A. Domains fused to either pUT18c or pKT25 for the BACTH assay. Hatched bars indicate transmembrane regions and functional domains are annotated according to the UniProt database (http://www.uniprot.org/uniprot/Q2G2U4 for WalK, http://www.uniprot.org/uniprot/Q2G2U1 for SaeS and http://www.uniprot.org/uniprot/Q2FVT1 for SpdC). B. β-galactosidase activities of DHT1 *E*. *coli* strains co-transformed with pUT18c containing either the full-length *spdC* coding sequence or a fragment encoding only the first 252 amino-acids of SpdC, and pKT25 derivatives carrying DNA fragments encoding the indicated proteins. Positive interaction results are shaded. ND: Not determined.

We focused our analysis on the WalK and SaeS histidine kinases. WalK has two transmembrane domains (amino acids 14–34 and 183–203), with an extracellular loop of 148 amino acid residues, a HAMP domain involved in signal transduction (204–256), a PAS domain (261–331) a PAC domain (314–378) and a histidine kinase domain (382–600; Fig 8A). In contrast, SaeS is a member of the intra-membrane sensing kinases [45], with two transmembrane domains (amino acids 9–29 and 40–60) separated by only ten amino acids, as well as a HAMP domain (61–114) and a histidine kinase domain (129–348; Fig 8A). We tested the interactions of SpdC with the N-terminal domains of WalK and SaeS containing the transmembrane regions (WalK_1-203_ and SaeS_1-64_, respectively). The truncated proteins were fused to the T25 domain of adenylate cyclase, and the resulting plasmids were co-transformed into *E*. *coli* strain DHT1 together with the pUT18c-*spdC* plasmid. As shown in Fig 8B, the first 203 amino acids of WalK are sufficient to allow stable interactions with SpdC. For SaeS, the N-terminal domain containing only the two transmembrane segments (SaeS_1-64_) did not lead to interaction with SpdC, but a longer fragment of the protein (SaeS_1-120_) gave rise to a stable interaction with SpdC and high β-galactosidase activity (Fig 8B). These results suggest that WalK and SaeS interact with SpdC through their transmembrane domains. The negative interaction results obtained with the truncated SaeS protein containing only the transmembrane domains suggest that since this kinase lacks an extracellular loop, the HAMP domain may be required for proper membrane insertion of the fusion protein.

In order to identify which domain of SpdC interacts with the WalK and SaeS kinases, we compared interactions with full-length SpdC (pUT18c-*spdC*) and a carboxy-terminal truncated SpdC consisting only of the eight transmembrane domains (SpdC_1-252_; pUT18c-*spdC*1-252). We noted self-interaction of SpdC following co-transformation of pKT25-*spdC* with either pUT18c-*spdC* or pUT18c-*spdC*1-252 (Fig 8B), indicating that the SpdC transmembrane domains are involved in these self-interactions. Similar results were obtained when testing interactions with WalK and SaeS, i.e. the transmembrane domains of SpdC are sufficient to allow interactions with the histidine kinases (Fig 8B).

Taken together, these results indicate that SpdC is a membrane-bound protein that interacts with itself and several histidine kinases through transmembrane domain contacts.

## Discussion

Abi-domain proteins constitute a large family whose functions are mostly unknown. SpdC was initially designated LyrA for Lysostaphin resistance A and identified by screening a *bursa aurealis* transposon mutant library for increased lysostaphin resistance [46]. An independent study aiming at characterizing proteins involved in the display of surface proteins led to the identification of three proteins, SpdA, SpdB and SpdC (Surface protein display A, B and C), playing a role in protein A levels at the staphylococcal cell surface [31]. These proteins share a similar structural organization with 6 to 8 transmembrane domains and an Abi-domain embedded within the hydrophobic region. An additional protein with a similar organization is encoded by the *S*. *aureus* genome, SAOUHSC_02256, but appears to have no role in controlling protein A levels [31].

We previously characterized the essential WalKR two-component system in *S*. *aureus* and highlighted its major role in controlling cell wall homeostasis [9, 47]. Transcriptome analysis revealed that *spdC* expression is positively controlled by WalKR [6]. We show here that the SpdC membrane protein and the WalK histidine kinase interact and that SpdC negatively controls WalKR activity and expression of WalKR-regulated genes. Interaction of the WalK histidine kinase with SpdC is specific since no interaction was seen with the other Abi-proteins (SpdA, SpdB and SAOUHSC_02256). Accordingly, we also showed that SpdC and WalK are both localized preferentially at the division septum.

Interestingly, an RNA-Seq analysis of a Δ*sdpC* mutant revealed that the expression of 107 genes varied compared to the parental strain. Among these, 24 (more than 20%) are controlled by the WalKR system. Since SpdC appears to negatively control WalKR activity by interacting with WalK, this septal localization is consistent with the previously suggested cell wall metabolism-related activation signal of the WalK histidine kinase in *Bacillus subtilis* [11, 48]. Indeed, in cocci, cell wall synthesis has been shown to exclusively occur at the division septum in an FtsZ-dependent manner [49], suggesting that a peptidoglycan metabolism related signal at the septum may relieve negative control of WalK activity by SpdC.

Histidine kinases often act as phosphoprotein phosphatases towards their associated response regulator. WalK was previously classified as a kinase/phosphatase «bifunctional sensor» [23] and the PAS domain of *Streptococcus pneumoniae* WalK plays a role in its phosphatase activity [50, 51]. In *S*. *aureus*, we have previously shown that WalK acts as a WalR phosphoprotein phosphatase upon entry into stationary phase in order to shut off WalR activity [6]. Interactions between SpdC and WalK can either interfere with signal perception by the sensor histidine kinase, inhibit its kinase activity or increase its phosphatase activity towards WalR, thus negatively controlling WalKR-dependent gene expression. SpdC is unlikely to directly regulate gene expression since it is a membrane protein lacking any typical DNA-binding domain.

To understand how the other SpdC-dependent genes were controlled (83 of the genes identified by RNA-Seq are not regulated by the WalKR system), we tested interactions of SpdC with the other *S*. *aureus* histidine kinases and found that it interacts with 10 of the 16 encoded in the genome. No obvious structural motifs or domain sequences were common to those that interacted with SpdC compared to those that did not. Two histidine kinases, WalK and SaeS, were chosen for further analysis of their interactions with SpdC. Our results indicate that the two transmembrane domains of WalK are sufficient to allow interaction with SpdC, whereas a greater amino-terminal fragment of SaeS was required, encompassing the HAMP domain. This result suggests that although the transmembrane domains of SaeS are likely involved in interactions with SpdC, a longer fragment may be necessary to ensure proper membrane insertion of the truncated protein. We also showed that a truncated form of SpdC, containing only the eight transmembrane domains, was sufficient for self-interaction and interaction with WalK and SaeS, indicating that transmembrane domains are involved in the interactions between SpdC and the histidine kinases. Interestingly, the only two *S*. *aureus* cytoplasmic histidine kinases which lack transmembrane domains, AirS and NreB, did not interact with SpdC under our conditions, in agreement with our results indicating transmembrane segments are involved in the interactions. The only other example of an Abi domain protein interacting with a histidine kinase is Abx1 of *Streptococcus agalactiae*, which interacts with the CovS kinase [25]. The two transmembrane domains of CovS were shown to be necessary and sufficient for these interactions [25].

At least fifteen genes belonging to the SaeSR regulon, including the *spl* operon, *sak*, the *hlgBC* operon and *chp* [7, 38–40], are also positively controlled by SpdC (Table 1), indicating that SpdC likely activates the SaeSR TCS, in contrast to its role in negatively controlling activity of the WalKR system. Since WalR controls *spdC* expression, this is consistent with our previous results showing that constitutive activation of WalR generates a signal leading to activation of the SaeSR TCS and a corresponding increase in SaeSR regulon expression [6].

The localization of SpdC at the division septum and its role in gene regulation through interactions with sensor kinases of two-component systems led us to speculate that SpdC may interfere with bacterial division sensing and impact cell wall metabolism. Accordingly, the Δ*spdC* mutant displays increased resistance against lysis when treated with lysostaphin, in agreement with the original phenotype characterized by transposon insertion [46]. Additionally, the absence of SpdC was reported to lead to increased cross wall abundance and thickness [31]. We tested sensitivity of the Δ*spdC* mutant to antibiotics targeting the cell wall. The Δ*spdC* mutant is highly sensitive to oxacillin and tunicamycin, but not to fosfomycin, which inhibits the first step of cell wall biosynthesis. In agreement with the sensitivity of the Δ*spdC* mutant to tunicamycin, which inhibits wall teichoic acid synthesis, the *spdC* gene was also identified as a candidate using a screen to identify synthetically lethal mutations with teichoic acid biosynthesis defects [52]. We have shown that expression of cell wall hydrolase genes is increased in the Δ*spdC* strain (*sceD*, *ssaA*, *lytM*, *atlA*). This may lead to increased cell wall degradation, which could explain the lowered resistance to oxacillin. This could also lead to the mutant’s increased sensitivity to tunicamycin. Indeed, teichoic acids are key elements for the proper localization of AtlA to the division septa, where cell wall biosynthesis takes place, since cell wall plasticity is essential for cell division [53]. In the presence of tunicamycin, the absence of wall teichoic acids results in a delocalized distribution of AtlA across the cell surface. Since *atlA* is more highly expressed in the Δ*spdC* stain, this could explain why this strain is more sensitive to tunicamycin.

Taken together with the strong links to the WalKR TCS, these results indicate that SpdC is involved in bacterial cell envelope homeostasis. The importance of the bacterial cell envelope in host-pathogen interactions cannot be over-emphasized: it is the first layer of contact between the bacterium and its host, containing an array of cell wall-linked or associated toxins and virulence factors, the first and major bacterial line of defense against threats from the host or environment, and is also both the target of choice for antibiotic treatment and the source of many antibiotic-resistance pathways. We have shown that SpdC is required for biofilm formation, an important step in *S*. *aureus* pathogenesis. We also showed that SpdC is a novel *S*. *aureus* virulence gene, required for the infectious process in a mouse septicemia model. The loss of virulence observed with the Δ*spdC* mutant may involve both its diminished capacity to form biofilms as well as the lowered expression of multiple virulence genes as shown by RNA-Seq analysis.

The regulatory mechanism mediated by SpdC remains to be determined. Abi-domain-containing proteins have been extensively studied in eukaryotes. They are involved in CAAX-protein maturation by cleaving the C-terminal AAX tripeptide after addition of an isoprenyl group on a cysteine, the last step consisting in methylation of the new C- terminus. These three modification steps are termed prenylation [54]. This post-translational maturation has a crucial role in maintaining cellular homeostasis by controlling the localization and activity of a large range of proteins. In particular, by adding a lipid group at the carboxy-terminal end of proteins, it favors their interactions with membranes, which have a high concentration of signaling molecules [55]. Prenylation has been recently described in prokaryotes and a geranyltransferase, IspA, has been identified in *S*. *aureus* [56]. Putative methyltransferase and CAAX-protease encoding genes (including *spdC*) are also present in the *S*. *aureus* genome. Deletion of the *ispA* gene has pleiotropic effects such as a growth defect, increased sensitivity to oxidative stress and an altered cell envelope [56]. Of note, the absence of IspA or SpdC both lead to increased cell wall antibiotic sensitivity. The RNA-Seq transcriptome analysis of the Δ*ispA* strain [56] shows similarities with that of the Δ*spdC* mutant. Indeed, one of the most regulated genes in both cases is *spa*, encoding the immunoglobulin G binding protein A. We also noted that 21 Φ13 genes are up-regulated in the Δ*ispA* mutant whereas we characterized 11 Φ13 genes up-regulated in the Δ*spdC* mutant, with several in common. These data suggest that SpdC and IspA could be involved in the same cellular pathway.

Interestingly, the transcriptome analysis of the Δ*ispA* mutant revealed modified expression of a large number of genes involved in regulatory circuits and particularly increased expression of the *walR*, *walH*, *walI* and *walJ* genes of the *wal* locus [56]. While no direct link between prenylation and Abi domain proteins has been shown in prokaryotes, there are several links with the WalKR system, either by physical interaction and negative control of activity, for SpdC, or at the transcriptional level for the IspA geranyltransferase.

This study identifies a membrane-bound protein with an Abi domain, SpdC, at the core of an interaction network that coordinates bacterial division with cell envelope metabolism and host interactions. Further studies are required in order to decipher the molecular mechanism and consequences of these interactions.

## Materials and methods

### Bacterial strains and growth media

*Escherichia coli* K12 strain DH5α (Invitrogen, Thermo Fisher Scientific, Waltham, MA) was used for cloning experiments. *Staphylococcus aureus* strain HG001 [57] was used for genetic and functional studies. Plasmids were first passaged through the restriction deficient *S*. *aureus* strain RN4220 before introduction into the HG001 strain. *E*. *coli* strains were grown in LB medium with ampicillin (100 μg/ml) added when required. *S*. *aureus* strains and plasmids used in this study are listed in Table 2. *S*. *aureus* strains were grown in Trypticase Soy Broth (TSB; Difco; Becton, Dickinson and Co., Franklin Lakes, NJ) supplemented with chloramphenicol (10 μg/ml) or erythromycin (1 μg/ml) as required. *E*. *coli* and *S*. *aureus* strains were transformed by electroporation using standard protocols [58] and transformants were selected on LB or Trypticase Soy Agar (TSA; Difco) plates, respectively, with the appropriate antibiotics. Expression from the P*cad* promoter was induced by adding cadmium chloride (CdCl_2_) at a final concentration of 0.25 μM. Expression from the Pspac promoter was induced by addition of isopropyl β-D-1-thiogalactopyranoside (IPTG).

**Table 2:** Strains and plasmids.

Strain or plasmid	Description	Source or reference
***E*. *coli* strains**		
DH5α	F^**-**^ Φ80*lac*ZΔM15 Δ(*lac*ZYA-*arg*F) U169 *rec*A1 *end*A1 *hsd*R17 (rK–, mK+) *pho*A *sup*E44 λ– *thi*-1 *gyr*A96 *rel*A1	Invitrogen Life Technology
DHT1	F^**-**^*glnV44*(AS) *recA1 endA1 gyrA96* (Nal^r^) *thi-1 hsdR17 spoT1 rfbD1 cya-854 ilv-691*::Tn*10*	[72]
***S*. *aureus* strains**		
RN4220	Restriction-deficient transformation recipient strain	[75]
HG001	NCTC 8325 *rsbU+*	[57]
ST1000	RN4220 P*spac-walRKHI*	[9]
ST1017	HG001 P*spac-walRKHI*	This study
ST1317	HG001 Δ*spdC*	This study
ST1375	HG001 Δ*spdC*/pMK4Pprot-*spdC*	This study
ST1386	HG001/pSD3-41 (*spdC*’-*lacZ*)	This study
ST1342	HG001/pOLSA-*spdC*	This study
**Plasmids**		
pMAD	Allelic exchange vector	[59]
pMAD*spdC*	pMAD derivative for *spdC* deletion	This study
pMK4Pprot	pMK4 derivative with the constitutive Pprot promoter for complementation	[60]
pMK4Pprot-*spdC*	pMK4Pprot derivative with the *spdC* gene	This study
pSA14	Vector for constructing transcriptional *lacZ* fusions	[62]
pSD3-41	pSA14 derivative carrying the *spdC* promoter region transcriptionally fused to *lacZ*	This study
pOLSA	Vector for expression of GFP protein fusions	[30]
pOLSA-*spdC*	pOLSA derivative containing the *spdC* gene translationally fused with GFP	This study
pKT25	BACTH vector designed to express a polypeptide fused at its N-terminal end with the adenylate cyclase T25 fragment; p15 ori	[35]
pUT18c	BACTH vector designed to express a polypeptide fused at its N-terminal end with the adenylate cyclase T18 fragment; ColE1 ori	

### DNA manipulations

Oligonucleotides used in this study were synthesized by Eurofins Genomics (Ebersberg, Germany) and their sequences are listed in Table 3. *S*. *aureus* chromosomal DNA was isolated using the MasterPure Gram-positive DNA purification Kit (Epicentre Biotechnologies, Madison, WI). Plasmid DNA was isolated using a QIAprep Spin Miniprep kit (Qiagen, Hilden, Germany) and PCR fragments were purified using the Qiaquick PCR purification kit (Qiagen). T4 DNA ligase and restriction enzymes, PCR reagents and Q5 high-fidelity DNA polymerase (New England Biolabs, Ipswich, MA) were used according to the manufacturer's recommendations. Nucleotide sequencing of plasmid constructs was carried out by Beckman Coulter Genomics (Danvers, MA).

**Table 3:** Oligonucleotides used in this study.

Name	Sequence	Description
***Construction of pMAD-spdC***		
OP375	TGCAGGATCCGATTGTTGTTCAGCACGTGT	Upstream fragment (*Bam*HI/*XmaI*)
OP376	AGCACCCGGGTATATGTAACCTCCATTAGGT	
OP377	AGCACCCGGGTAACAAAGCGCTTGCTAGTA	Downstream fragment (*XmaI*/*Nco*I)
OP378	ATTCTCCATGGGATTACATAAATATGGGAGGC	
***Construction of pMK4Pprot-spdC***		
OP404	ACTGGATCCTATTTTGCTTGTTACCTAATGGAGG	*spdC* coding sequence (*Bam*HI/*Sal*I)
OP405	AGCGTCGACTTTGTTATTATTTGTTTTTATCTG	
***Construction of pSD3-41 (pSA14-PspdC)***		
OSA512	CTTCTGCAGAGCATTTCCCCCTCTTATTTATGTG	*spdC* upstream region (*Pst*I/*Bam*HI)
OSA513	GTTGGATCCTATATGTAACCTCCATTAGGTAACAAGC	
***Construction of pOLSA-spdC***		
OSA417	CTTGTCGACTAATGGAGGTTACATATAATGAAGAAC	*spdC* coding sequence (*Sal*I/*Xma*I)
OSA404	TGCCCGGGTTTGTTTTTATCTGAAGATTGTTCTTCAG	
***Construction of BACTH plasmids***		
OSA385	GTTGGATCCCATGAAGAACAATAAAATTTCTGGTTTTCAATGGG	*spdC* (*saouhsc*-02611) (*Bam*HI/*Kpn*I)
OSA386	AGCGGTACCTTTGTTATTATTTGTTTTTATCTGAAG	
OSA379	AGGGGATCCCATGCAAAAATTCAAAGACTTTTTTTACG	*spdA* (*saouhsc*-1900) (*Bam*HI/*Kpn*I)
OSA380	TGAGGTACCTATGAAAGGTTATCTCAAAATTATCTCC	
OSA383	GGAGGATCCCGTGGAAAATGAAAAAAAGAAATACACG	*spdB* (*saouhsc*-02587) (*Bam*HI/*Kpn*I)
OSA384	ACTGGTACCCCCGACCTCTTTATCTACGCATAAATA	
OSA381	GAGGGATCCCATGACAAGATTATGGGCATCATTGC	*saouhsc*-02256 (*Bam*HI/*Kpn*I)
OSA382	AGCGGTACCATCCGTGTGTGATTCGTTTTTTTTATTATGG	
OSA363	TCGGATCCCATGAAGTGGCTAAAACAACTACAATCCC	*walK* (*saouhsc*-00021) (*Bam*HI/*Kpn*I)
OSA364	AGGGTACCTATGCTCCTTATTATTCATCCC	
OSA488	AGAGGATCCCATGACGGCATACAAACCTTATAGAC	*yesM* (*saouhsc*-00185) (*Bam*HI/*Eco*RI)
OSA489	ATAGAATTCTCATCATCACAAATAACTACC	
OSA490	ATCGGATCCCATGTTATTACTTGAGCGTGTAGG	*lytS* (*saouhsc*-00230) (*Bam*HI/*Eco*RI)
OSA491	CATGAATTCTGATTAATGCTTTCATATTTATTCC	
OSA492	TATGGATCCCATGAATAATTTGAAATGGGTAGC	*graS* (*saouhsc*-00666) (*Bam*HI/*Eco*RI)
OSA493	TCAGAATTCAGTAACAAAACGCATGTTTAAAATGAC	
OSA397	GAGGATCCCATGGTGTTATCAATTAGAAGTCAAATCATTATTGC	*saeS* (*saouhsc*-00714) (*Bam*HI/*Kpn*I)
OSA398	TAGGTACCAATCGGATTATGACGTAATGTCTAATTTGTG	
OSA494	GGAGGATCCCATGAAATTTTTAAAAGATACTTC	*desK* (*saouhsc*-01313) (*Bam*HI/*Eco*RI)
OSA495	TTTGAATTCTCTGCAATAATTAAAGATGTCATGC	
OSA496	ATGGGATCCCATGACAAAACGTAAATTGCGCAATAAC	*arlS* (*saouhsc*-01419) (*Bam*HI/*Eco*RI)
OSA497	AAAGAATTCACTTTGATTGACGTCTCAGTCATG	
OSA482	GTTGGATCCCATGATGAGCCGGCTAAATAGTG	*ssrB* (*saouhsc*-01585) (*Bam*HI/*Eco*RI)
OSA483	GTTGAATTCTAACTATATTCAATTTTATTCTGG	
OSA480	AAGGGATCCCATGAAGTTTCACCACCGCTTAATG	*phoR* (*saouhsc*-01799) (*Bam*HI/*Eco*RI)
OSA481	TACGAATTCTGTTACCACTTTAATTTTTATTC	
OSA498	GAGGGATCCCATGGAACAAAGGACGCGACTAGC	*airS* (*saouhsc*-01981) (*Bam*HI/*Eco*RI)
OSA499	TTTGAATTCATGGGTTATCTCCTTAAATCAAGC	
OSA484	TGAGGATCCCATGAACCACTACATTAGAACAATTGG	*vraS* (*saouhsc*-02099) (*Bam*HI/*Kpn*I)
OSA485	ATCGGTACCAAACAATACTTTAATCGTCATACG	
OSA401	TTGGATCCCATGATATTAATGTTTACAATACCAGC	*agrC* (*saouhsc*-02264) (*Bam*HI/*Kpn*I)
OSA402	CAGGTACCTCCTTATGGCTAGTTGTTAATAATTTCAAC	
OSA500	GGTGGATCCCATGTCAAACACTGAATCGCTAAAC	*kdpD* (*saouhsc*-02314) (*Bam*HI/*Eco*RI)
OSA501	CTTGAATTCTCAATATTTTAGATTGCATTATACG	
OSA502	TGGGGATCCCATGTTTAAAACACTCTATGCTAG	*hssS* (*saouhsc*-02644) (*Bam*HI/*Eco*RI)
OSA503	AAAGAATTCTTCAGGAGGTAGAGATTAAAGTG	
OSA504	GGTGGATCCCATGATTAATGAGGACAGTATACAG	*nreB* (*saouhsc*-02676) (*Bam*HI/*Kpn*I)
OSA505	TTTGGTACCCCAATGTATGTTTCAAATTGGAATG	
OSA506	GGTGGATCCCATGACCTTTCTTAAAAGTATTACTC	*braS* (*saouhsc*-02955) (*Bam*HI/*Eco*RI)
OSA507	ACTGAATTCATTGAAAGTTTTTATTCATCTGG	
OSA385	GTTGGATCCCATGAAGAACAATAAAATTTCTGGTTTTCAATGGG	*spdC* 1–252 (*Bam*HI/*Kpn*I)
OSA520	AATGGTACCGATAATTAAGCTTAAACCAATGTATCC	
OSA363	TCGGATCCCATGAAGTGGCTAAAACAACTACAATCCC	*walK* 1–203 (*Bam*HI/*Kpn*I)
OSA518	AATGGTACCCGCTATAAAGAATCCTAGGATGAC	
OSA397	GAGGATCCCATGGTGTTATCAATTAGAAGTCAAATCATTATTGGC	*saeS* 1–120 (*Bam*HI/*Kpn*I)
OSA517	TTCGGTACCTTCGGATTTAATTTGATTCATTTGTTGC	
OSA397	GAGGATCCCATGGTGTTATCAATTAGAAGTCAAATCATTATTGGC	*saeS* 1–64 (*Bam*HI/*Kpn*I)
OSA519	AATGGTACCTATAAGTGGATTAATAAAAATACTAC	
***qRT-PCR experiments***		
OSA161	ACGTGGATAACCTACCTATAAGACTGGGAT	*16s rRNA*
OSA162	TACCTTACCAACTAGCTAATGCAGCG	
OSA391	GCAGTAGGATACATTGGTT	*spdC*
OSA392	CAGCCTCAGTATGATTAGTT	
OSA203	AACAGCACCAACGGATTAC	*atlA*
OSA204	CATAGTCAGCATAGTTATTCATTG	
OSA127	ATCAAATACAACATTAACTGTCGTTGATGC	*sdrD*
OSA128	CATGTTTTGCAGTCGCAATTGTTTCACC	
OP321	TGAGTCAGACATTAGGATAC	*hlgC*
OP322	TTGTTGTTCTACTTCACTTAC	
OP291	AAGTGCTAACCTATTGTCAGAAG	*spa*
OP292	TCGTCTTTAAGGCTTTGGATG	
OSA218	AGCGAACAGTAATAACTACCAATG	*lytM*
OSA219	CGATGCCACCAGACATACG	
OSA231	ACAGGTACTAATGGAGCAGAC	*sceD*
OSA232	TGTGGTTGTTGAGTTTGAGC	
OSA138	GTGTACTGTGCATACGATGGTAATGATGC	*walR*
OSA139	CGTTACATAGTCATCTGCACCTAGTTCTA	

### Plasmid and mutant construction

For construction of the Δ*spdC* mutant strain, two 800 bp DNA fragments were generated by PCR using oligonucleotide pairs OP375/OP376 and OP377/OP378, respectively (see Table 3), corresponding to the DNA regions located immediately upstream and downstream from the *spdC* gene. These DNA fragments were cloned in tandem in two consecutive steps, between the *Bam*HI and *Nco*I restriction sites of the pMAD vector. The resulting plasmid was introduced by electroporation into *S*. *aureus* and transformants were selected at 30°C on TSA plates containing erythromycin (1 μg/ml) and 5-bromo-4-chloro-3-indolyl-β-D-galactopyranoside (X-Gal, 100 μg/ml). Integration and excision of the plasmid were then performed as previously described [59], yielding mutant strain ST1317 (Δ*spdC*). A complementation plasmid pMK4Pprot-*spdC* was constructed by cloning the entire *spdC* coding sequence (amplicon OP404/OP405) in plasmid pMK4Pprot, under the control of the constitutive P*prot* promoter [60]. The plasmid was introduced into the ST1317 Δ*spdC* mutant, generating the ST1375 complemented strain. Expression of the *walKRHI* in strain HG001 was placed under the control of the IPTG-iducible Pspac promoter by Φ80α phage transduction [61] using strain ST1000 (RN4220 Pspac*walRKHI*; [9]) as a donor and strain HG001 as the recipient, yielding strain ST1017 (HG001 Pspac*walRKHI*).

### β-Galactosidase assays

Plasmid pSA14 [62] is a derivative of shuttle vector pMK4 [63], carrying a promoterless *E*. *coli lacZ* gene and was used to construct transcriptional *lacZ* reporter fusions. The *spdC* promoter region was amplified by PCR using oligonucleotides OSA512/OSA513 (see Table 3) and cloned between the *Pst*I/*Bam*HI restriction sites of the pSA14 vector, yielding plasmid pSD3-41 (Table 2).

For β-galactosidase assays in *S*. *aureus*, strain ST1386 carrying the *spdC*’-*lacZ* fusion was grown in TSB at 37°C and cells were harvested by centrifuging 2 ml culture samples (2 min; 5,400 x *g*). Assays were performed as previously described [21] and β-galactosidase specific activities expressed as Miller units mg^−1^ protein [64]. Protein concentrations were determined using the Bio-Rad protein assay (BioRad, Hercules, CA) [65].

### Total RNA extraction

Strains were grown in TSB, supplemented with IPTG when specified, at 37°C with aeration until OD_600nm_ = 1. Cells were pelleted by centrifugation (2 min, 20,800 x *g*) and immediately frozen at -20°C. RNA extraction was then performed as previously described [66], followed by DNaseI treatment with the TURBO DNA-free reagent (Ambion, Austin, TX) in order to eliminate residual genomic DNA.

### cDNA synthesis and quantitative real time PCRs (qRT-PCRs)

cDNA synthesis was carried out as previously described [47]. Oligonucleotides were designed with the BEACON Designer 7.91 software (Premier Biosoft International, Palo Alto, CA) in order to synthesize 100–200 bp amplicons (see Table 3). Quantitative real-time PCRs (qRT-PCRs), critical threshold cycles (CT) and *n*-fold changes in transcript levels were performed and determined as previously described using the SsoFast EvaGreen Supermix (Bio-Rad, Hercules, CA) and normalized with respect to 16S rRNA whose levels did not vary under our experimental conditions [47]. All assays were performed using quadruplicate technical replicates, and repeated with three independent biological samples.

### RNA-Seq transcriptome analysis

Three independent biological replicates were used for RNA-Seq analysis of the parental HG001 and ΔspdC strains. Strains were grown in TSB until OD_600nm_ = 1. Total RNA was isolated as described above, and 7 μg were treated using the MicrobExpress kit (Ambion, Austin, TX) in order to remove rRNA. The rRNA depleted fraction was used for construction of strand specific single end cDNA libraries using the Truseq Stranded Total RNA sample prep kit according to the manufacturer’s instructions (Illumina, San Diego, CA). Libraries were sequenced using an Illumina Hiseq2000 sequencer (multiplexing 6 samples in one lane) according to the manufacturer’s instructions (Illumina, San Diego, CA).

Sequences were demultiplexed using the Illumina alignment and sequence analysis pipeline (GERALD, included in CASAVA version 1.7) giving FASTQ formatted reads. Reads were cleaned by removing adapter and low quality sequences using an in-house program (https://github.com/baj12/clean_ngs). Only sequences with a minimum length of 25 nucleotides were considered for further analysis. Bowtie (version 0.12.7, -m50—chunkmbs 400 -a—best -q -e50) [67] was used for alignment with the reference *Staphylococcus aureus* subsp. *aureus* genome (gi|88193823). Only uniquely aligning reads where considered for counting. HTseq-count (version 0.5.4p5, parameters: -m intersection-nonempty, -s yes, -t CDS -I locus_tag) was used for counting genes [68].

Statistical analysis was performed with R version 3.0.2 [69] and DESeq2 version 1.2.10 [70]. Data were first normalized with DESeq2 and the default parameters. Dispersion estimation and statistical testing were performed using the Generalized Linear Model with default parameters. Independent filtering was performed with default parameters to exclude transcripts with very low count values. Raw *P*-values were then adjusted according to the Benjamini and Hochberg procedure [71] and transcripts were considered differentially expressed when their adjusted *P*-value was lower than 0.05.

### BACTH protein interaction assays

For testing protein interactions using the Bacterial Adenylate Cyclase Two-Hybrid System (BACTH), genes encoding the proteins of interest were cloned into plasmids pKT25 and pUT18c leading to translational fusions with the T25 or T18 domains of the *Bordetella pertussis* adenylate cyclase [35]. DNA fragments corresponding to the coding sequences were amplified by PCR using chromosomal DNA from strain HG001 and specific oligonucleotide pairs (see Table 3). Fragments were digested with *Bam*HI and *Eco*RI or *Kpn*I (indicated in Table 3) for cloning into plasmids pKT25 or pUT18c. The resulting plasmids were co-transformed into *E*. *coli* strain DHT1 [72] to detect protein-protein interactions and transformants were selected on kanamycin (50 μg/ml) for pKT25 derivatives and ampicillin (100 μg/ml) for pUT18c derivatives.

The resulting strains carrying combinations of pKT25 and pUT18c derivatives were tested for cyclic AMP-dependent activation of *lacZ* expression. For tests on plates, strains were grown in LB liquid medium supplemented with ampicillin (100 μg/ml) and kanamycin (50 μg/ml). Overnight cultures were then spotted on LB-agar plates containing IPTG (0.5 mM), ampicillin (100 μg/ml), kanamycin (50 μg/ml), and X-Gal (100 μg/ml). Plates were incubated for 24 H at 30°C and examined for appearance of the characteristic blue color indicative of β-galactosidase activity through X-Gal hydrolysis. Quantitative β-galactosidase assays were performed on exponentially growing *E*. *coli* liquid cultures. Cells were grown in LB with IPTG (0.5 mM), ampicillin (100 μg/ml) and kanamycin (50 μg/ml) at 30°C under aeration until OD_600nm_ = 1 and assays performed on SDS/chloroform permeabilized cells as previously described [64]. Enzymatic activities are represented relative to negative and positive controls, respectively a strain carrying the empty pKT25 and pUT18c vectors (activity = 0, arbitrary unit), and a strain with the pKT25-zip and pUT18c-zip plasmids [35] (activity = 1000, arbitrary unit).

### Subcellular localization of SpdC

The pOLSA plasmid was used to produce a fluorescent SpdC-GFP fusion protein [30]. The translational fusion was constructed by PCR amplification using HG001 chromosomal DNA and oligonucleotide pair OSA417/OSA404 (Table 3). The amplicon was cloned into pOLSA between the *Sal*I and *Xma*I restriction sites, allowing transcription from the P*cad* promoter and production of the SpdC-GFP fusion protein.

Subcellular protein localization of SpdC was performed in *S*. *aureus* HG001 transformed with pOLSA-*spdC*. Fluorescence microscopy was carried out on cells grown in liquid cultures in TSB supplemented with CdCl_2_ (0.25 μM) to induce gene fusion expression. When cells reached OD_600nm_ ≈ 1.5 (exponential growth phase), they were harvested and concentrated 20 times in PBS. Cell suspensions were mixed with Vectashield mounting media (Vector Laboratories, Burlingame, CA) and used for microscopic observations with a Nikon Eclipse E600. Images were acquired with a Nikon DXM1200F Digital Camera. ImageJ software was used for quantifying fluorescence (http://imagej.nih.gov/ij/index.html; [73]). Fluorescence ratios were calculated by measuring fluorescence at the division septa divided by the fluorescence at the lateral wall after subtracting background fluorescence. Quantification was performed for 33 cells and two independent biological replicates and plotted using GraphPad Prism (GraphPad Software, San Diego, CA; http://www.graphpad.com).

### Sensitivity to compounds targeting the cell envelope

The HG001, Δ*spdC* and Δ*spdC*/pMK4Pprot-spdC strains were grown overnight at 37°C with aeration in TSB medium, with chloramphenicol (10 μg/ml) when required. Bacterial suspensions diluted from 10^−2^ to 10^−7^ were spotted (3 μl) onto TSA plates with the indicated antibiotic concentrations and incubated for 15 hours at 37°C.

### Protein preparation and Western blotting

*S*. *aureus* whole cell lysates were prepared as previously described [47]. Briefly, 5 ml of a cell culture grown to stationary phase were harvested by centrifugation (10 min; 3,000 x *g*), resuspended in 2X Laemmli SDS sample buffer (0.2 ml) and heated at 99°C for 10 min. Supernatants containing SDS-soluble proteins were collected following centrifugation (10 min; 20,800 x *g*), and used for further analysis. Cell wall extracts were prepared from 50 ml of the same cultures. Cells were pelleted and resuspended in 4 ml of digestion buffer (50 mM Tris-HCl pH 8, 145 mM NaCl, 30% sucrose, 160 ng/ml DNaseI, 250 μg/ml lysostaphin) and incubated for 60 min at 37°C. Supernatants corresponding to cell wall extracts were then harvested by centrifugation (10 min; 3,000 x *g*). Protein extracts were separated by SDS-PAGE on a 12% polyacrylamide gel, followed by Coomassie Brilliant Blue staining to verify that the quality and quantity of loaded extracts was equivalent for the different strains. For immunoblotting experiments, protein extracts were transferred to a nitrocellulose membrane after SDS-PAGE using a semi-dry blotter (Bio-Rad, Hercules, CA) and the following buffer: 25 mM Tris, 192 mM glycine, 20% ethanol. The LytM protein was detected using a purified polyclonal rabbit antibody [74] and horseradish peroxidase-coupled anti-rabbit secondary antibodies (Zymed, ThermoFisher, Waltham, MA) and the Pico chemiluminescence Western blot kit (Pierce, ThermoFisher, Waltham, MA). Detection of Spa was carried out directly using the secondary antibodies. Purified *Staphylococcus aureus* Protein A was obtained from Sigma-Aldrich (St. Louis, MO).

### Lysostaphin-induced lysis assays

Strains were grown in TSB, with 10 μg/ml chloramphenicol for the complemented strain, at 37°C under aeration. When the OD_600nm_ reached 1, bacteria were harvested by centrifugation, (10 min; 3000 x *g*), washed in PBS, and resuspended in the same volume of PBS (control) or PBS containing 200 ng/ml lysostaphin followed by incubation at 37°C. Lysis was monitored by measuring the decline in OD_600nm_ over time and indicated as a percentage of the initial OD (measured OD_600nm_/initial OD_600nm_).

### Biofilm formation assay

Biofilm assays were performed by growing cells in PVC microtiter plates (200 μl per well) in TSB with 0.75% glucose and 3.5% NaCl. After 24 h static growth at 37°C, adherent biomass was rinsed twice with PBS, air dried, stained with 0.1% crystal violet for 15 min, resuspended in ethanol-acetone (80:20) and quantified by measuring OD_595nm_, normalized to the OD_600nm_ of each culture (growth rates for the different strains were the same).

### Murine infection experiments

Seven-week-old female RjOrl:SWISS mice (Centre d’Elevage Roger Janvier, Le Genest-St.-Isle, France) were inoculated intravenously with the *S*. *aureus* HG001 parental strain and the otherwise isogenic Δ*spdC* mutant. Groups of seven mice were infected with 5.10^7^ cfu per mouse in 0.2 ml. Survival was monitored daily over 9 days post-infection and three independent experiments were carried out. Virulence of the complemented strain could not be carried out since we have shown that in vivo, in the absence of selection pressure, the complementation plasmid was lost over the assay period. Indeed, after nine days post-infection with the ST1375 complemented strain, animals were sacrificed and the kidneys removed and homogenized for determination of bacterial CFU (total and chloramphenicol resistant) per kidney, revealing that 97% of the bacteria had lost the pMK4Pprot-*spdC* complementation plasmid.

### Ethics statement

Animal experiments were conducted at the Institut Pasteur in compliance with French legislation (Decree N° 2001–464 05/29/01) and European Union guidelines on handling of laboratory animals:

(http://ec.europa.eu/environment/chemicals/lab_animals/index_en.htm).

Animals were sacrificed by increasing carbon dioxide concentrations. Protocols were approved by the Institut Pasteur ethics committee (Authorization N° 2013–0032).

### Accession numbers

The complete RNA-Seq dataset was deposited in the EMBL European Nucleotide Archive (accession number PRJEB11849) and is accessible at the following URL:

http://www.ebi.ac.uk/ena/data/view/PRJEB11849

## Supporting information

S1 FigGrowth curves of *S*. *aureus* HG001 and the Δ*spdC* mutant.Bacterial cultures were grown overnight, inoculated in TSB at a calculated OD_600nm_ of 0.05 and incubated at 37°C with aeration. Optical densities were followed over a 6-hour period. Results are shown as the mean and standard deviation of three independent growth curves. Doubling times (http://www.doubling-time.com/compute.php) were calculated during the exponential growth phase (between 90 min and 214 min) and gave identical values of 32 min for each strain. Strains: HG001 (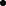); Δ*spdC* (○).(TIFF)

S2 Fig Expression of *spdC*’-*lacZ* during growth. *Staphylococcus aureus* strain ST1386 cells carrying plasmid pSD3-41 (*spdC*’-*lacZ* fusion) were grown in TSB at 37°C and harvested at the indicated OD_600 nm_ values by centrifuging 2 ml culture samples (2 min; 5,400 x *g*). β-galactosidase assays were then performed as previously described [21] and β-galactosidase specific activities expressed as Miller units mg^−1^ protein [64]. Protein concentrations were determined using the Bio-Rad protein assay (BioRad, Hercules, CA) [65]. Results are shown as the mean and standard deviation of three independent experiments. The background value for the strain carrying the pSA14 plasmid with the promoterless *lacZ* gene was less than two Miller units mg^−1^ protein under the same conditions.
(TIFF)

S3 Fig SpdC does not affect Triton-induced autolysis. Bacteria were grown in TSB at 37°C with aeration until OD_600 nm_ ≈ 1, pelleted (10 min; 5,400 x *g*), resuspended in phosphate buffered saline (PBS) with Triton X-100 (0.1%), and incubated at 37°C with aeration. Lysis was determined as the decrease in OD_600 nm_ over time and indicated as a percentage of the initial OD (measured OD_600 nm_ / initial OD_600 nm_). Results are shown as the mean and standard deviation of three independent experiments. Strains: HG001 (■); Δ*spdC* (▲).
(TIFF)

S4 Fig Biofilm detachment assays. Biofilm assays were performed by growing cells in PVC microtiter plates (200 μl per well) in TSB with 0.75% glucose and 3.5% NaCl. After 24 h static growth at 37°C, biofilm cultures were treated with DNAseI (5 μg/ml), proteinase K (1 μg/ml), or sodium metaperiodate (25 mM) for two hours before washing and staining of biofilms. The remaining adherent biomass was rinsed twice with PBS, air dried, stained with 0.1% crystal violet for 15 min, resuspended in ethanol-acetone (80:20) and quantified by measuring OD_595nm_, normalized to the OD_600nm_ of each culture. The values represent the mean of two biological replicates and seven technical replicates and standard deviations are indicated.
(TIFF)
