# Supplementary figures and images for: SpdC, a novel virulence factor, controls histidine kinase activity in Staphylococcus aureus
Source: PLoS Pathog. 2018 Mar 15;14(3):e1006917. doi: 10.1371/journal.ppat.1006917 (PMC5854430; doi:10.1371/journal.ppat.1006917)

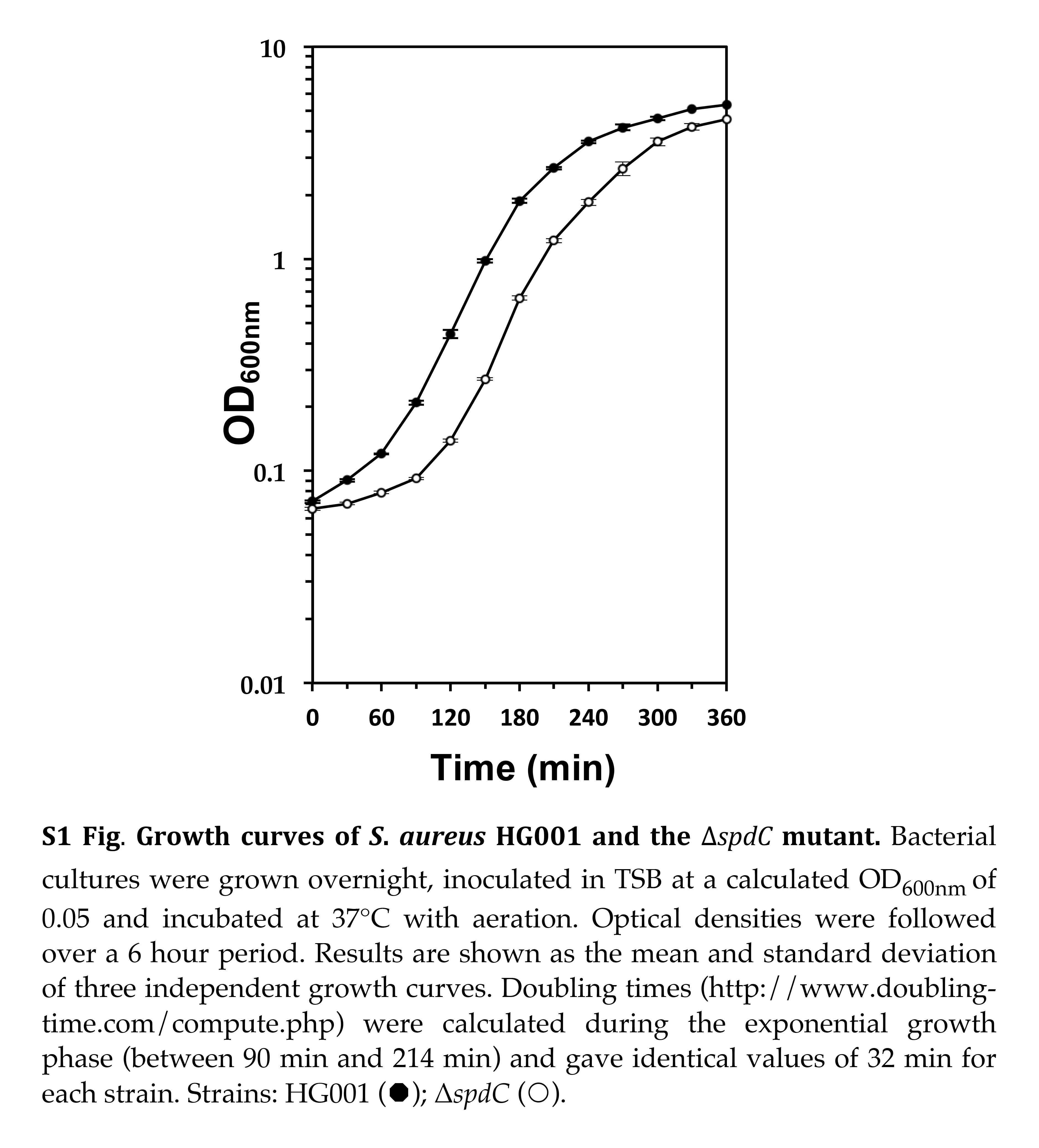

Supplement: S1 Fig — Bacterial cultures were grown overnight, inoculated in TSB at a calculated OD600nm of 0.05 and incubated at 37°C with aeration. Optical densities were followed over a 6-hour period. Results are shown as the mean and standard deviation of three independent growth curves. Doubling times (http://www.doubling-time.com/compute.php) were calculated during the exponential growth phase (between 90 min and 214 min) and gave identical values of 32 min for each strain. Strains: HG001 (); ΔspdC (○). (TIFF) [file ppat.1006917.s001.tiff]

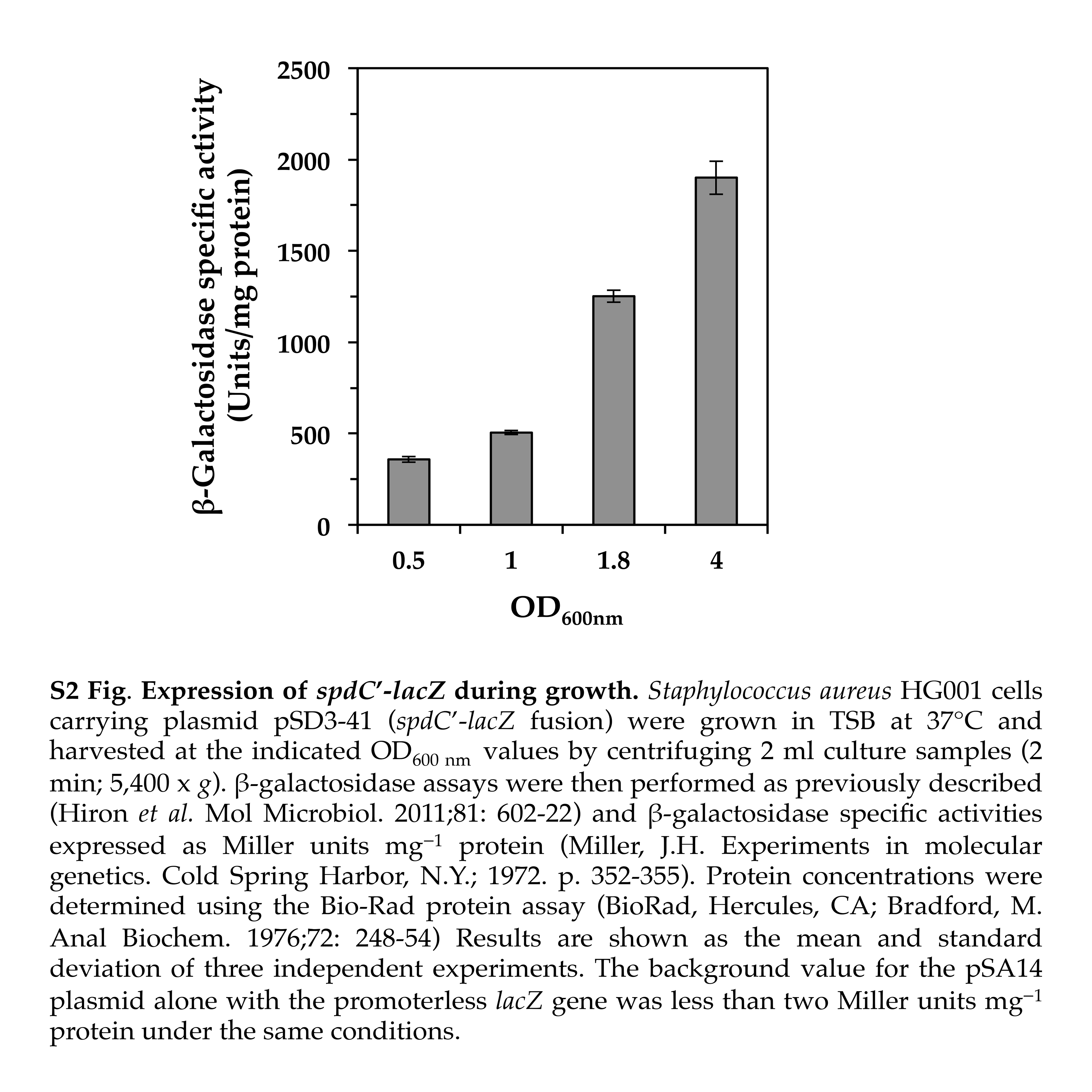

Supplement: S2 Fig — Staphylococcus aureus strain ST1386 cells carrying plasmid pSD3-41 (spdC’-lacZ fusion) were grown in TSB at 37°C and harvested at the indicated OD600 nm values by centrifuging 2 ml culture samples (2 min; 5,400 x g). β-galactosidase assays were then performed as previously described [21] and β-galactosidase specific activities expressed as Miller units mg−1 protein [64]. Protein concentrations were determined using the Bio-Rad protein assay (BioRad, Hercules, CA) [65]. Results are shown as the mean and standard deviation of three independent experiments. The background value for the strain carrying the pSA14 plasmid with the promoterless lacZ gene was less than two Miller units mg−1 protein under the same conditions. (TIFF) [file ppat.1006917.s002.tiff]

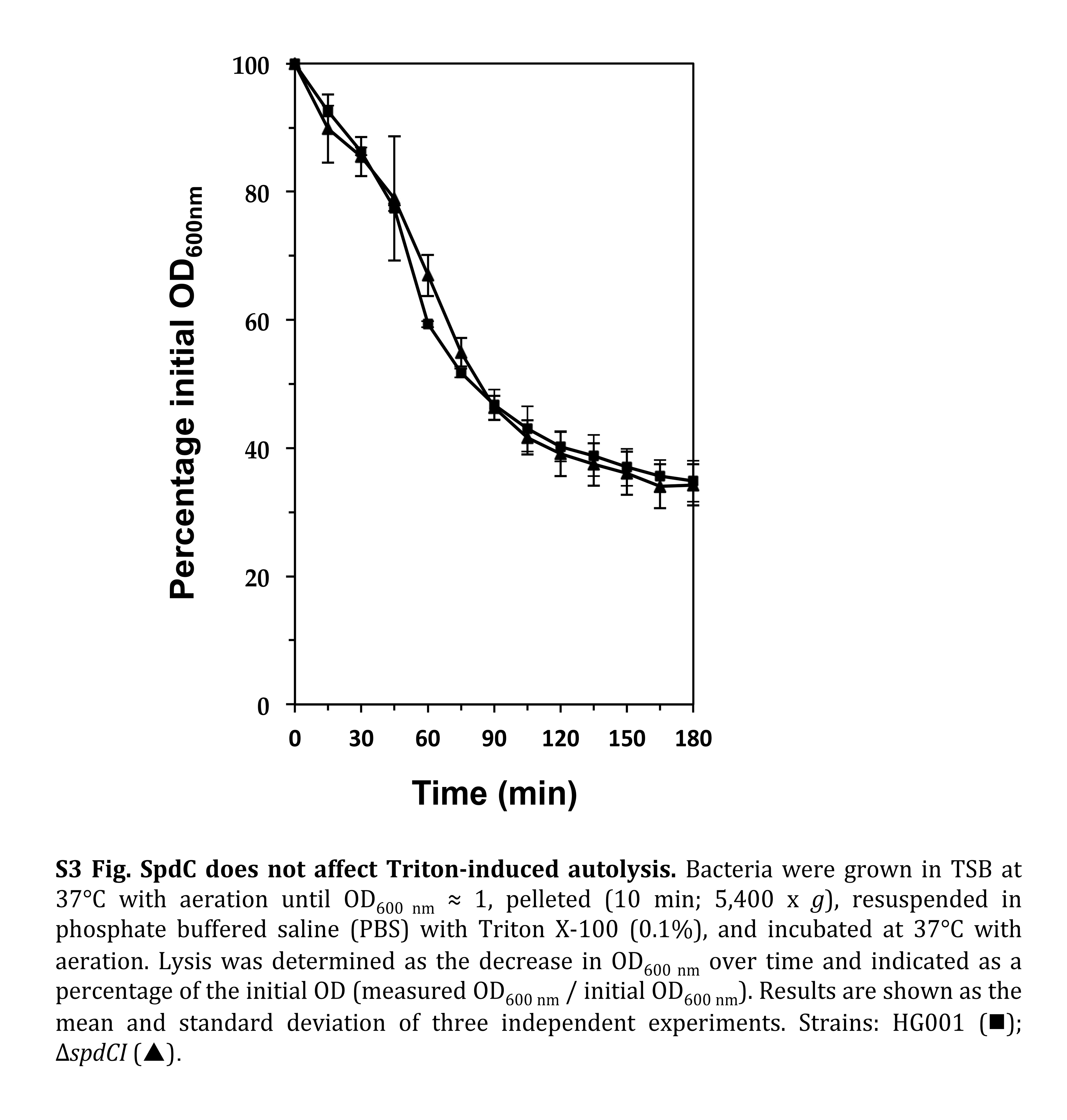

Supplement: S3 Fig — Bacteria were grown in TSB at 37°C with aeration until OD600 nm ≈ 1, pelleted (10 min; 5,400 x g), resuspended in phosphate buffered saline (PBS) with Triton X-100 (0.1%), and incubated at 37°C with aeration. Lysis was determined as the decrease in OD600 nm over time and indicated as a percentage of the initial OD (measured OD600 nm / initial OD600 nm). Results are shown as the mean and standard deviation of three independent experiments. Strains: HG001 (■); ΔspdC (▲). (TIFF) [file ppat.1006917.s003.tiff]

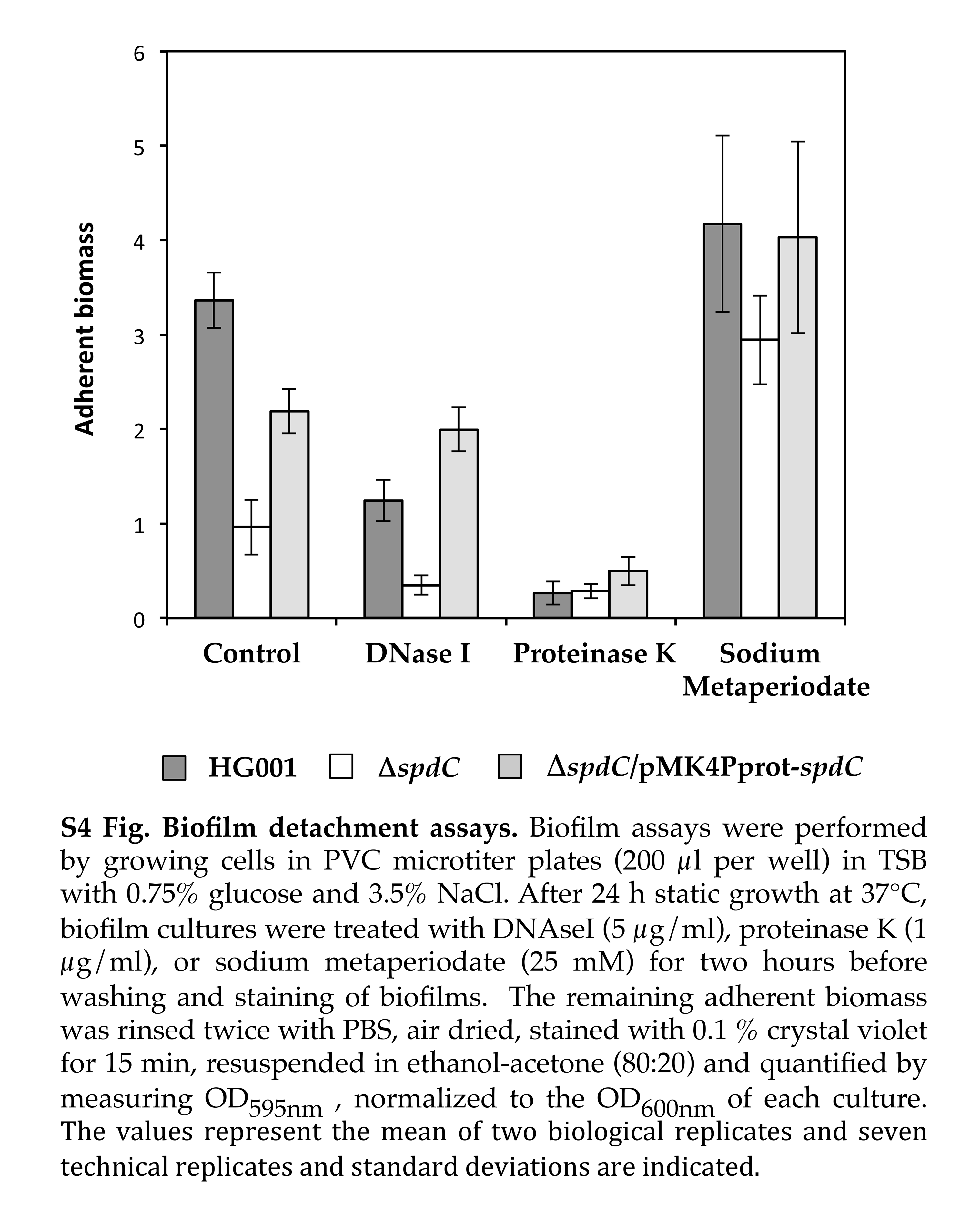

Supplement: S4 Fig — Biofilm assays were performed by growing cells in PVC microtiter plates (200 μl per well) in TSB with 0.75% glucose and 3.5% NaCl. After 24 h static growth at 37°C, biofilm cultures were treated with DNAseI (5 μg/ml), proteinase K (1 μg/ml), or sodium metaperiodate (25 mM) for two hours before washing and staining of biofilms. The remaining adherent biomass was rinsed twice with PBS, air dried, stained with 0.1% crystal violet for 15 min, resuspended in ethanol-acetone (80:20) and quantified by measuring OD595nm, normalized to the OD600nm of each culture. The values represent the mean of two biological replicates and seven technical replicates and standard deviations are indicated. (TIFF) [file ppat.1006917.s004.tiff]
